# Leveraging Large Language Models in the delivery of post-operative dental care: a comparison between an embedded GPT model and ChatGPT

**DOI:** 10.1038/s41405-024-00226-3

**Published:** 2024-06-12

**Authors:** Itrat Batool, Nighat Naved, Syed Murtaza Raza Kazmi, Fahad Umer

**Affiliations:** Section of Dentistry, Department of Surgery, Aga Khan University Hospital, Karachi, Pakistan

**Keywords:** Health care, Dentistry

## Abstract

**Objective:**

This study underscores the transformative role of Artificial Intelligence (AI) in healthcare, particularly the promising applications of Large Language Models (LLMs) in the delivery of post-operative dental care. The aim is to evaluate the performance of an embedded GPT model and its comparison with ChatGPT-3.5 turbo. The assessment focuses on aspects like response accuracy, clarity, relevance, and up-to-date knowledge in addressing patient concerns and facilitating informed decision-making.

**Material and methods:**

An embedded GPT model, employing GPT-3.5-16k, was crafted via GPT-trainer to answer postoperative questions in four dental specialties including Operative Dentistry & Endodontics, Periodontics, Oral & Maxillofacial Surgery, and Prosthodontics. The generated responses were validated by thirty-six dental experts, nine from each specialty, employing a Likert scale, providing comprehensive insights into the embedded GPT model’s performance and its comparison with GPT3.5 turbo. For content validation, a quantitative Content Validity Index (CVI) was used. The CVI was calculated both at the item level (I-CVI) and scale level (S-CVI/Ave). To adjust I-CVI for chance agreement, a modified kappa statistic (K*) was computed.

**Results:**

The overall content validity of responses generated via embedded GPT model and ChatGPT was 65.62% and 61.87% respectively. Moreover, the embedded GPT model revealed a superior performance surpassing ChatGPT with an accuracy of 62.5% and clarity of 72.5%. In contrast, the responses generated via ChatGPT achieved slightly lower scores, with an accuracy of 52.5% and clarity of 67.5%. However, both models performed equally well in terms of relevance and up-to-date knowledge.

**Conclusion:**

In conclusion, embedded GPT model showed better results as compared to ChatGPT in providing post-operative dental care emphasizing the benefits of embedding and prompt engineering, paving the way for future advancements in healthcare applications.

## Introduction

Machine learning (ML) is a subset of Artificial Intelligence (AI) that allows computer systems to analyze data, identify patterns, and make intelligent decisions without explicit programming [1]. The integration of AI in healthcare has become imperative as the current healthcare infrastructure is ill-prepared to manage the increased clinical workload, resulting in extended patient wait times, burnout among healthcare professionals, and added strain on the healthcare system [2]. Moreover, it can contribute to more accurate diagnosis, treatment planning, improved patient interaction, and the automation of various tasks within the field of dentistry [3]. Amongst the many tasks that AI performs, its role in patient interaction is increasingly significant, bringing about improvements in communication and overall healthcare experience [4].

Large Language Model (LLM) such as Open AI’s ChatGPT (Generative Pre-trained Transformer) within the realm of Natural Language Processing (NLP) is a subset of ML that analyzes and responds to human language input in a conversational manner [5]. These models leverage large-scale pre-training on diverse datasets, learning contextual relationships and generating human-like text. Moreover, they can be embedded for specific applications or a variety of language-related tasks [6].

An AI chatbot is a computer program that utilizes LLM to interpret user input in the form of text or speech and generate contextually relevant responses [5]. Such chatbot models can offer patients swift and convenient access to precise and trustworthy information cost-effectively and with round-the-clock availability [7]. While these conversational bots exhibit certain capabilities, they still necessitate oversight from surgeons and their healthcare teams [8]. In the field of medicine various chat-bots have been evaluated such as the study by Lim et al. which analyzed the performance of LLM models for myopia care and study by Dwyer et al. that assessed the conversational chatbot performance post hip arthroscopy surgery [9, 10]. However, a comprehensive assessment of large language models in answering patients’ post-operative questions in the field of dentistry has not yet been thoroughly evaluated.

Dental procedures require patients to adhere to specific post-operative instructions diligently. These instructions, if followed strictly, can significantly influence the outcome of the procedures and the patient’s overall experience [11]. However, ensuring that patients not only receive but also comprehend and adhere to these instructions has been a longstanding challenge for dental practitioners. This is where the innovative integration of chatbots into dental care can become invaluable [8].

This paper aims to evaluate the performance of an embedded GPT model (conversational chatbot) in providing dental post-operative instructions to patients and its comparison with ChatGPT-3.5 turbo. The assessment focuses on aspects like response accuracy, clarity, relevance, and up-to-date knowledge in addressing patient concerns and facilitating informed decision-making.

This study addresses the need for innovative solutions in dental care by evaluating the effectiveness of embedded GPT models in providing post-operative instructions to patients, aiming to improve patient comprehension and adherence. By comparing the performance of ChatGPT-3.5 turbo, this research aims to assess the viability of advanced conversational chatbots in enhancing patient experience and decision-making in dentistry.

## Materials and methods

Although ethical approval for the conduct of study was not required, the research has been done within local ethical frameworks and regulations, as outlined in the Declaration of Helsinki. Creating a custom conversational chatbot involved a meticulous process where we carefully tailored the embedded GPT chatbot through distinct phases. GPT-3.5-16k version was selected after the registration on GPT trainer website (https://gpt-trainer.com/), that provides access to customized features. The embedded model was configured to adhere to the following base prompt*:*

*“I want you to roleplay as AI Assistant for dental problems. You will provide me with answers from the given context. The answers should be as precise as possible in no more than 50 words. Do not refer to empirical remedies. Do not answer any irrelevant questions unrelated to dental problems and do not answer any medical health problems. Never break character*.”

Additionally, to ensure that the chatbot is well informed, it was embedded by providing relevant dental post-operative instructions scraped from the internet in a Word file format (Supplementary Note 1). The embedding stores all the necessary information for the LLM model to search and produce relevant outputs in response to the user’s query. These vectors, containing embeddings, are stored in an index within a vector database, providing a structured organization of information, a process known as Retrieval Augmentation Generation (RAG) [12]. We intentionally set the temperature of the embedded model at a low level to mitigate incidence of hallucinations.

Likewise, ChatGPT-3.5 turbo (https://chat.openai.com/) underwent prompt engineering, utilizing the same base prompt but without the inclusion of the embedded file. The default temperature setting of the model was not changed.

In the following text, for the convenience of readers, the embedded GPT 3.5-16k model (conversational chatbot) and OpenAI’s ChatGPT 3.5 turbo (prompt engineered) will be referred to as “embedded GPT model” and “ChatGPT” respectively.

A questionnaire was generated representing 40 real life questions (Supplementary Note 2) from four specialties of dentistry, namely: Oral and Maxillofacial Surgery, Operative Dentistry & Endodontics, Periodontics and Prosthodontics. The questions were designed to reflect the types of inquiries posed by the patients following dental procedures with the aim of providing a representative sample of questions the chatbot would encounter in a real-world setting. The formulated questions were validated by a panel of experts belonging to the respective specialties. The responses to these questions were then generated via the embedded GPT model and ChatGPT (Supplementary Note 2).

The recorded responses were shared with the specialists via Google Forms. This research engaged experts from various institutions, opting for a web-based platform due to its cost-effectiveness and efficiency in facilitating participation from diverse locations. Consent was obtained prior to the completion of questionnaire by the dental professionals. A purposive sampling method was employed for the selection of participants. The sampling strategy aimed to ensure that the participants possess a specialized postgraduate degree in the respective specialty of dentistry as part of inclusion criteria. Considering the recommendations, the number of experts for content validation should be at least six and does not exceed ten [13]. Therefore, a total of 36 participants (9 belonging to each four specialties of dentistry i.e., Oral & Maxillofacial Surgery, Operative Dentistry & Endodontics, Periodontics and Prosthodontics) formed the study sample. To eliminate bias in grading, the experts were blinded to the responses generated via both models. The responses generated via embedded GPT model and ChatGPT were assigned a special code “A” or “B” respectively (Supplementary Note 2).

**Fig. 1:**
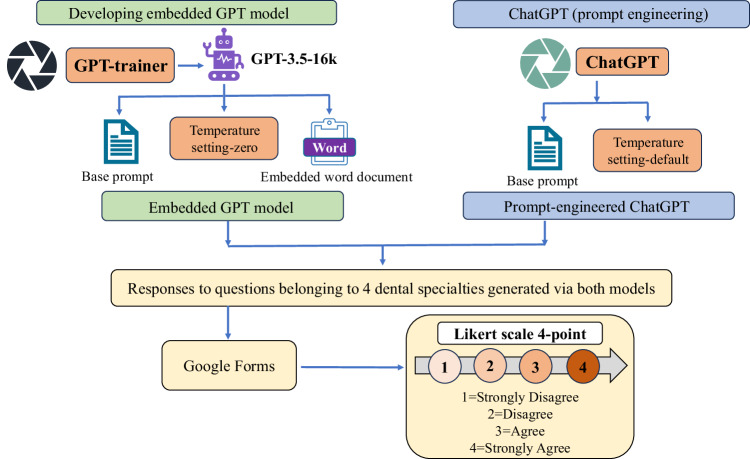
Graphical representation of methodology. An embedded GPT model was created by selecting GPT-3.5-16k on GPT-trainer. A base prompt was provided along with embedding and the temperature setting was maintained at zero. The responses to the questions were generated via embedded GPT model and were compared with the prompt-engineered chatGPT to which the same base prompt was provided but without embedding and the default temperature setting was retained as well. The responses were then evaluated by a series of experts (dental professionals) on a 4-point Lkert scale.

The methodology is graphically presented in Fig. 1.

### Content validation

For content validation of the responses generated via both models, a quantitative Content Validity Index (CVI) was used. The CVI was calculated both at the item level (I-CVI) and scale level (S-CVI/Ave) (Table 1). Experts were asked to rate each response based on content validity indicators such as relevance, clarity, accuracy, and up-to-date knowledge, using a 4-point Likert scale:

$$1={{{{{\rm{Strongly\; disagree}}}}}},2={{{{{\rm{Disagree}}}}}},3={{{{{\rm{Agree,\;}}}}}}4={{{{{\rm{Strongly\; agree}}}}}}$$

**Table 1:** Operational definition and interpretation of item-level and scale-level content validity index.

CVI indices	Definition	Formula	Interpretation
I-CVI (item-level content validity index)	The proportion of experts giving a rating of 3 or 4 to a response (item)	I-CVI = no. of experts rating a response as 3 or 4/total number of experts	I-CVI = 0.78 or more (response is acceptable) I-CVI = 0.70–0.77 (response requires revision) I-CVI < 0.70 (response is eliminated)
S-CVI/Ave (scale-level content validity index)	The average of the I-CVI scores for all responses on the scale judged by all experts	S-CVI = sum of I-CVI scores/number of items	S-CVI = 0.90 or more (overall excellent content validity) S-CVI > 0.8 (acceptable content validity)

For ease of interpretation, the ordinal scale on the instrument was dichotomized into two i.e., experts in agreement (score 3 and 4) versus disagreement (score 1 and 2). Item-level content validity index (I-CVI) was then calculated for each item by dividing the number of experts in agreement (or disagreement) by the total number of experts. Moreover, to adjust I-CVI for chance agreement, a modified kappa statistic (K*) was computed. To ascertain the average number of items scoring 3 or 4 amongst the evaluators, an average scale level content validity index (S-CVI/Ave) was reported by calculating the mean of I-CVI values (sum of I-CVI scores divided by total number of items).

## Results

A panel of 36 experts, nine from each domain participated in the content validation process of the responses generated via the GPT embedded model and ChatGPT. Each expert evaluated 20 responses (10 responses per GPT model) in terms of relevance, accuracy, clarity, and up-to-date knowledge.

**Fig. 2:**
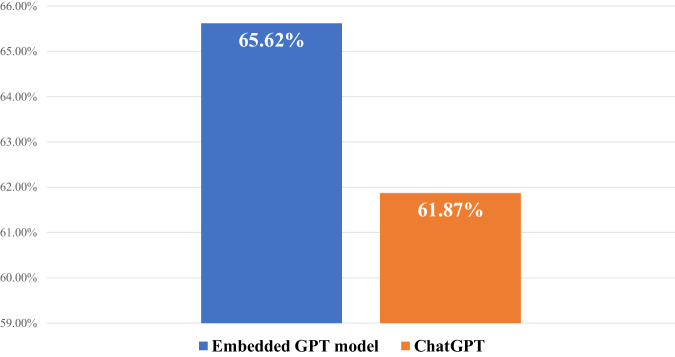
Overall content validity of responses generated via both models. The embedded GPT model performed better with 65.62% responses in the acceptable range overall compared to chatGPT with 61.87% acceptable responses.

The overall content validity of responses generated via GPT embedded model and ChatGPT was 65.62% and 61.87% respectively (Fig. 2).

**Fig. 3:**
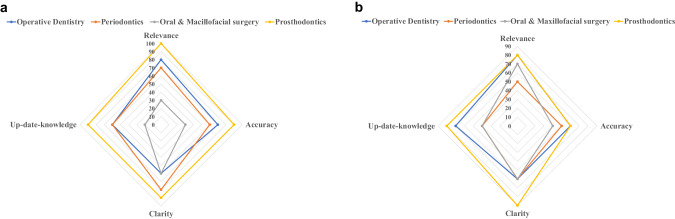
Specialty-wise distribution of responses with acceptable I-CVI (values = 0.78 or more) generated via both models. **a** Percentage of responses with acceptable I-CVI values generated via embedded GPT model. **b** Percentage of responses with acceptable I-CVI values generated via ChatGPT.

The specialty-wise percentage of responses with acceptable I-CVI values generated via both models are presented in Fig. 3a, b.

For acceptable responses in all four domains, the kappa statistic revealed an inter-rater reliability between 0.75 to 1 representing excellent agreement amongst the evaluators. The individual I-CVI and kappa values for each item generated via both models are presented in Supplementary Notes 3–6.

The specialty-wise content validity (I-CVI and S-CVI) of the responses generated via both models are presented below and in Tables 2–5.

**Table 2:** Content validity of the responses generated via embedded GPT model in terms of I-CVI.

Specialty	Relevance (*n* = 10)	Clarity (*n* = 10)	Accuracy (*n* = 10)	Up-date-knowledge (*n* = 10)	Frequency (%)
Operative Dentistry	8	6	7	6	27 (67.5)
Periodontics	7	8	6	6	27 (67.5)
Oral & Maxillofacial Surgery	3	6	3	2	14 (35)
Prosthodontics	10	9	9	9	37 (92.5)
Frequency (%)	28 (70%)	29 (72.5%)	25 (62.5%)	23 (57.5%)	**65.62%**^a^

Number of responses with acceptable I-CVI (between 0.78–1).

^a^Overall content validity (acceptability). The numbers in bold represent the overall content validity score.

**Table 3:** Content validity of the responses generated via embedded GPT model in terms of S-CVI.

Specialty	Relevance	Clarity	Accuracy	Up-date-knowledge
Operative Dentistry	0.822^a^	0.811^a^	0.789^b^	0.756^b^
Periodontics	0.80^a^	0.87^a^	0.77^b^	0.76^b^
Oral & Maxillofacial Surgery	0.644^b^	0.72^b^	0.644^b^	0.644^b^
Prosthodontics	0.944^a^	0.867^a^	0.833^a^	0.844^a^

S-CVI of the responses.

^a^Acceptable content validity (S-CVI = 0.8 or greater).

^b^Unacceptable content validity (S-CVI < 0.8).

**Table 4:** Content validity of the responses generated via chatGPT in terms of I-CVI.

Specialty	Relevance (*n* = 10)	Clarity (*n* = 10)	Accuracy (*n* = 10)	Up-date-knowledge (*n* = 10)	Frequency (%)
Operative Dentistry	8	6	6	7	27 (67.5)
Periodontics	5	6	5	4	20 (50)
Oral & Maxillofacial Surgery	7	6	4	4	21 (52.5)
Prosthodontics	8	9	6	8	31 (77.5)
Frequency (%)	28 (70%)	27 (67.5%)	21 (52.5%)	23 (57.5%)	**61.87%**^a^

Number of responses with acceptable I-CVI (between 0.78–1).

^a^Overall content validity (acceptability). The numbers in bold represent the overall content validity score.

**Table 5:** Content validity of the responses generated via chatGPT in terms of S-CVI.

Specialty	Relevance	Clarity	Accuracy	Up-date-knowledge
Operative Dentistry	0.856^a^	0.811^a^	0.800^a^	0.811^a^
Periodontics	0.756^b^	0.70^b^	0.69^b^	0.62^b^
Oral & Maxillofacial Surgery	0.722^b^	0.733^b^	0.700^b^	0.700^b^
Prosthodontics	0.867^a^	0.844^a^	0.800^a^	0.856^a^

S-CVI of the responses.

^a^Acceptable content validity (S-CVI = 0.8 or greater).

^b^Unacceptable content validity (S-CVI < 0.8).

### Embedded GPT model

#### Operative Dentistry & Endodontics

The content validity (acceptability) of the individual responses in terms of relevance, accuracy, clarity and up-to-date knowledge was 67.5% i.e., 27 out of 40 responses showed an acceptable I-CVI (between the range of 0.78–1).

The overall content validity of the responses revealed acceptable results in terms of relevance and clarity i.e., S-CVI=0.82 and 0.81 respectively. However, regarding the accuracy and up-to-date knowledge of the responses, the values were not satisfactory with S-CVI=0.78 and 0.75 respectively.

#### Periodontics

In periodontics, 27 out of 40 responses (67.5%) showed an acceptable I-CVI. The overall content validity of the responses revealed acceptable results in terms of relevance and clarity i.e., S-CVI=0.80 and 0.87 respectively. However, regarding the accuracy and up-to-date knowledge of the responses, the values were not satisfactory with S-CVI=0.77 and 0.76 respectively.

#### Oral & Maxillofacial Surgery

In oral surgery, only 14 out of 40 responses (35%) showed an acceptable I-CVI. The overall content validity of the responses revealed unsatisfactory results in terms of relevance S-CVI=0.644, clarity S-CVI=0.72, accuracy S-CVI=0.644, and up-to-date knowledge S-CVI=0.644.

#### Prosthodontics

In prosthodontics, 37 out of 40 responses (92.5%) showed an acceptable I-CVI with an inter-rater reliability between 0.75 to 1 showing excellent agreement amongst the evaluators.

The overall content validity of the responses revealed acceptable results in terms of relevance S-CVI=0.944, clarity S-CVI=0.867, accuracy S-CVI=0.833 and up-to-date knowledge S-CVI=0.844.

### ChatGPT

#### Operative Dentistry & Endodontics

The content validity (acceptability) of the individual responses in terms of relevance, accuracy, clarity and up-to-date knowledge was 67.5% i.e., 27 out of 40 responses showed an acceptable I-CVI (between the range of 0.78–1).

The overall content validity of the responses revealed satisfactory results in terms of relevance S-CVI=0.856, clarity S-CVI=0.811, accuracy S-CVI=0.800 and up-to-date knowledge=S-CVI 0.811.

#### Periodontics

In periodontics, 20 out of 40 responses (50%) showed an acceptable I-CVI. The overall content validity of the responses revealed unsatisfactory results in terms of relevance S-CVI=0.756, clarity S-CVI=0.70, accuracy S-CVI=0.69 and up-to-date knowledge S-CVI=0.62.

#### Oral & Maxillofacial Surgery

In oral surgery, 21 out of 40 responses (52.5%) showed an acceptable I-CVI. The overall content validity of the responses revealed unsatisfactory results in terms of relevance S-CVI=0.722, clarity S-CVI=0.733, accuracy S-CVI=0.700, and up-to-date knowledge=S-CVI 0.700.

#### Prosthodontics

In prosthodontics, 31 out of 40 responses (77.5%) showed an acceptable I-CVI. The overall content validity of the responses revealed acceptable results in terms of relevance S-CVI=0.867, clarity S-CVI=0.844, accuracy S-CVI=0.800, and up-to-date knowledge=S-CVI 0.856.

## Discussion

The current healthcare system is struggling with challenges such as professional burnout and prolonged patient waiting times, resulting in increased workload and additional burden on the healthcare system [14]. In this regard, Large Language Models (LLMs), specifically AI chatbots can facilitate patient interaction by offering a promising solution to deliver timely and accurate information to patients [15]. While previous studies have explored the utility of LLMs in medicine [6] few studies have explored their application in different domains of dentistry [16, 17]. Furthermore, the existing studies primarily focused on aspects unrelated to the utility of LLM models in post-operative patient care and did not employ a quantitative analysis for content validation. Hence, there was a clear need for additional research in this specific domain.

This study investigated the impact of embedding a GPT model (RAG) on its performance and its comparison with ChatGPT with a keen focus on response accuracy, clarity, relevance, up-to-date knowledge. Accuracy was assessed because inaccurate information can lead to misunderstandings by patients, potentially impacting their recovery [18]. Evaluating the relevance ensured that the model-generated responses are directly applicable to the patient’s situation. Up to date knowledge was assessed as ChatGPT has its last training cut-off in 2021, so it cannot retrieve any new data beyond that date [19].

The results of the study revealed a superior performance for the embedded GPT model, surpassing ChatGPT with an accuracy of 62.5% and clarity of 72.5%. In contrast, the responses generated via ChatGPT achieved slightly lower scores, with an accuracy of 52.5% and clarity of 67.5%. However, both models performed equally well in terms of relevance and up-to-date knowledge. Remarkably both LLM models faltered in the specialty of Oral & Maxillofacial Surgery; this could be attributed to lack of specific and detailed information in the training dataset related to oral surgery procedures which may have hindered the model’s ability to provide comprehensive responses. Moreover, the nuanced nature of oral surgery topics may demand a higher level of domain specific context, which generic LLMs may not possess to the required extent.

Despite this, the embedded model exceeded expectations, performing admirably overall. The success of the model is attributed to not only embedding but also to the low temperature setting. Given the documented instances of hallucination in GPT models, precautionary measures were taken during the study [20]. Prompt engineering was implemented as a very specific base prompt was used for both models, and a conservative temperature setting was applied to limit the length of responses as well as to mitigate the risk of hallucinations. These measures were incorporated to enhance the reliability of the findings and to ensure that the responses generated by the models remained aligned with the intended context. Moreover, it is interesting to note that advance prompting techniques like role definition have only been used in two studies in dentistry previously [21, 22].

In the healthcare domain, the dissemination of misleading information can have severe consequences; this phenomenon has been elucidated in studies by Rahimi et al. and Deiana G et al. [23, 24]. Nevertheless, it is crucial to highlight that in our study, which incorporated embedding and utilization of low-temperature settings in the GPT model, no responses containing misleading information were generated.

The strengths of the study lie in its robust research design, which incorporated effective masking and randomization, and involved evaluations conducted by a group of 36 specialists. Moreover, a quantitative analysis utilizing content validation index was employed, distinguishing it from studies relying on surrogate measures [25]. Moreover, considering both indices i.e., I-CVI and S-CVI ensured a more thorough evaluation. This is crucial as the S-CVI, being an average score, can be influenced by outliers [26]. The novelty of this study lies in crafting an embedded model specifically designed for dentistry. Additionally, the comparative analysis offers valuable insights into the impact of embedding, enriching our understanding of model effectiveness [27].

Like any scientific endeavor, our study has its limitations. We utilized only chatGPT and embedded GPT model via their Application Programming Interface, meaning the findings might not be applicable to other Language Models. Additionally, it could have been beneficial to customize the Chat GPT model weights for our specific task. However, this was impractical due to the extensive computational resources required for such fine-tuning, which were not at our disposal. Instead, we opted for Retrieval Augmentation Generation (RAG), a method we believe to be novel in our context, as it has not been previously employed in similar studies. Furthermore, our use of a CVI scoring index for subject expert assessment, while not validated specifically for Language Model Models, offers potential advantages over the Likert scale commonly used in similar studies [28].

Further, limitations of the study include the constrained performance of the embedded model due to the limited information sourced from the internet during its training, potentially affecting its ability to address specific nuances in dental care. Moreover, the probabilistic nature of GPT models necessitates an evaluation of their consistency, acknowledging the challenge of assessing it due to resource constraints, particularly for embedded GPT models requiring tokens for generating responses [29]. Additionally, the study employed a subjective evaluation of content by specialists, which may yield varied outcomes based on individual expertise. The possible criticism to this paper can be that questions provided to the GPT models were not from actual patient. To mitigate this concern, we utilized four experts to validate the questions in order to enhance the generalizability.

While chatGPT is readily accessible to patients, the question arises: would they opt for a customized chatbot tailored to their specific needs? The study underscores that the efficacy of patient-accessible chatbots is enhanced through customization, achieved via prompt engineering and embedding, demonstrating that involving domain experts in their development improves utility and potentially results in superior performance for patients compared to the generic versions.

In considering the areas of future research, it would be worthwhile to explore enhancements in the fine-tuning process [30]. Specifically, investigating methodologies to enrich the training data with a more diverse range of patient queries and scenarios could contribute to a more adaptable model. Furthermore, exploring the integration of technologies such as reinforcement learning, or context aware models could enable the model to dynamically adjust its questioning strategy based on the evolving conversation resembling a more natural and interactive exchange with users. Another avenue of research could be assessing the practical effectiveness and acceptance to patients and their overall satisfaction of interacting with a deployed chatbot.

## Conclusion

The embedded GPT model showed better results in terms of accuracy and clarity as compared to ChatGPT in dental care context emphasizing the benefits of embedding and prompt engineering, paving the way for future advancements in healthcare applications.

### Supplementary information

Supplementary Note 1

Supplementary Note 2

Supplementary Note 3

Supplementary Note 4

Supplementary Note 5

Supplementary Note 6

Description of Additional Supplementary Files

## Data Availability

The data that support the findings of this study are available from the corresponding author, Dr. Fahad Umer upon reasonable request. Further the data which was used for Retrieval augmentation is supplied as supplementary material.
