# Supplementary material for: Leveraging Large Language Models in the delivery of post-operative dental care: a comparison between an embedded GPT model and ChatGPT
Source: BDJ Open. 2024 Jun 12;10:48. doi: 10.1038/s41405-024-00226-3 (PMC11169374; doi:10.1038/s41405-024-00226-3)
Supplement: Supplementary file 1 — Supplementary Note 1 [file 41405_2024_226_MOESM1_ESM.pdf]

Dental Post-Operative Instructions

After tooth extraction, it’s important for a blood clot to form to stop the bleeding and begin the healing process. That’s why we ask you to bite on a gauze pad for 45 minutes after the appointment. If the bleeding or oozing persists, insert another gauze pad and bite firmly for another 30 minutes. You may have to do this several times.

- Do not rinse or spit for 24 hours after surgery.
- Keep your fingers and tongue away from the socket or surgical area.
- Use ice packs on the surgical area (side of face) for the first 48 hours; apply ice 20 minutes on and 10 minutes off. (Note: Bags of frozen peas work well.)
- For mild discomfort, take Tylenol® or ibuprofen every 3–4 hours.
- For severe pain, use the medication prescribed to you.
- Drink plenty of fluids. (Do not use a straw—this creates suction in the mouth that could cause complications.)
- We suggest that you do not smoke for at least 5 days after surgery.
- Avoid strenuous activity and do not exercise for at least 3–4 days after surgery. After that, be careful: your regular caloric and fluid intake have been reduced, so you may get light-headed, dizzy, or weak.
- If the muscles of the jaw become stiff, the use of warm moist heat to the outside of your face over the spots that are stiff will relax these muscles.
- After the first post-operative day, use a warm salt water rinse following meals for the first week to flush out particles of food and debris that may lodge in the surgical area. (Mix ½ teaspoon of salt in a glass of warm water. Mouthwash can be added for better taste.)
- Your diet should consist mainly of soft, easily swallowed foods and cool drinks. Avoid anything that might get stuck in your teeth, so no seeds, nuts, rice, popcorn, or similar foods.

NSTRUCTIONS FOLLOWING ORAL SURGERY

***** EMERGENCIES – DO NOT HESITATE TO CALL OUR OFFICE AT ANY TIME IF IN DOUBT REGARDING THE FOLLOWING INSTRUCTIONS OR THE CONDITION OF THE PATIENT *****

Keep fingers and tongue away from tooth socket or operative site.
Do not rinse your mouth for 24 hours.

SMOKING
Do not smoke for 72 hours following surgery.

EATING
Do not rinse your mouth for 24 hours. It is permissible to drink lukewarm or cool liquids immediately, but do not use a straw. Soft foods can be taken as soon as tolerated. A regular diet can usually resume after 5 days.

BLEEDING
It is normal for the saliva to be lightly streaked with blood for a few days. If profuse ongoing bleeding is present, fold gauze into a firm, thick wad and place directly onto the socket or against the operative site and maintain steady pressure for 30 minutes. The less frequently the gauze is changed, the better. Do not spit out. Do not chew the gauze. In an emergency, a tea bag moistened in lukewarm water may be substituted for gauze. Do not rinse your mouth until 12 hours after the bleeding has stopped.

SWELLING
Considerable swelling may be expected in certain cases, reaching its maximum in 48-72 hours, then decreasing in 4-5 days. An ice pack can be applied outside the face next to the site of surgery, 20 minutes on and 20 minutes off, for the first 24 hours. This is not mandatory, however it may help to relieve discomfort and minimize swelling.

PAIN
For simple types of surgery, over the counter pain medication used as necessary should relieve discomfort. For more severe pain, we will prescribe medication to be taken as directed. Do not take any other pain medication with these tablets unless you have been specifically directed to do so. Do not drink any alcoholic beverages while taking these tablets for pain. Since many of these prescription drugs cause drowsiness, do not take them if you are driving a car, operating machinery, or doing anything requiring concentration.

ANTIBIOTICS
When antibiotics are prescribed they must be taken as directed. Continue taking the antibiotics until the prescription runs out unless you break out in a rash or have an adverse reaction (e.g. rash). In this case, stop taking the medication and inform our office.

ORAL HYGIENE
Provided that no bleeding is evident, begin brushing 24 hours after surgery. Rinse your mouth with lukewarm salt water {½ teaspoon salt in a glass of water} after each meal and brush your teeth using a soft toothbrush and toothpaste. Avoid brushing the surgical site for the first four days, then brush very gently to remove food debris. Do not rinse your mouth until the day after your surgery.

DISCOLORATION
Bruising of the skin occasionally occurs. It usually disappears in 7-10 days.

IMPACTED AND UNERUPTED TEETH

***** The removal of impacted teeth is different than the removal of erupted teeth. Your post-operative care is the same as that listed above. However, the following conditions may occur, none of which is unusual. *****

- The operated side may swell considerably.
- Trismus (tightness of the jaw muscles) may occur, making it difficult to open your mouth.
- You may have a sore throat or pain when you swallow.
- You may have numbness or tingling of the lip or tongue on the operated side. This condition is rarely permanent, but it may persist for several weeks or months.
- Adjoining teeth may ache temporarily.

- There may be an opening where the tooth was removed. The opening will gradually fill in.
- There may be a slight fever for 24-48 hours. If the fever persists, please call our office.

UNUSUAL CONDITIONS

- Fever over 101° F/39° C.
- Severe swelling after the third post-operative day.
- Severe, bright red bleeding that you cannot control by following the directions on the reverse of this page.
- Rash, difficulty breathing, severe vomiting, or any other suspected reaction to your medication.
- Prolonged or severe pain which is not relieved after several doses of the pain medication.

Post-Operative Instructions: Dental Implants

FOR THE REMAINDER OF THE DAY:

- Do not spit. Use a tissue to wipe your mouth as needed, or swallow your saliva.
- Do not use a drinking straw. Drink straight from the cup.
- Do not smoke.
- Keep fingers and tongue away from the surgical area.

Spitting, the use of a straw, smoking, and poking the surgical area can dislodge the blood clot that is forming and will cause bleeding from the area. Also, smoking can increase the chances of an infection.

BLEEDING:

Some minor bleeding is expected after dental implant surgery. It will usually subside quickly and stop within an hour or two after surgery. A little oozing is normal and may persist for several hours.

- Keep gauze on the surgical area with some biting pressure for 30–45 minutes.
- If all else fails, call the office.

SWELLING:

Most patients will experience some swelling after surgery in the mouth. It may be mild or severe and is different for every patient. The swelling may increase for the first 24–48 hours before it starts to go away. It may last for several days. Some bruising may also develop on the face.

DIET:

You may start with non-abrasive foods such as mashed potatoes, pasta, cottage cheese, soup, or scrambled eggs as soon as the local anesthetic wears off.

You may resume a regular diet as soon as you feel up to it; however, please try not to chew directly on the implant sites.

ORAL HYGIENE:

You may start rinsing tomorrow, very gently, with some warm salt water (1 tsp of salt in 8 ounces of warm water). You may brush your teeth per usual, though be very careful near the surgical sites.

SUTURES:

Unless told otherwise, the sutures used during your procedure are dissolvable and will fall out on their own. This generally occurs in the first week after surgery, but timing varies from 4–10 days.

BONE GRAFT MATERIAL:

If your surgeon placed bone graft material at the time the dental implant was placed, you might notice some gritty material in your mouth.

This is nothing to worry about and should subside within a day or two.

WILL I BE ABLE TO SEE THE IMPLANT?

Depending on the location and stability of the implant being placed, your surgeon may opt to bury the implant under the gum tissue. In time, you will return and have the implant uncovered so the implant can be restored.

If stability is optimal, the second stage procedure can be bypassed, and an attachment is placed that protrudes through the gum tissue, which will be visible. Once the bone has healed, the restoring dentist will be able to access the implant without the need for further surgery.

WHEN CAN THE CROWN BE PLACED?

A dental crown can be attached to a dental implant once the implant has fused to the bone. This is a process called osseointegration.

The length of time the bone must heal varies from case to case and will be discussed with your surgeon. The length of time needed for osseointegration is a function of the bone quality, quantity, and implant stability at the time of placement.

MEDICATIONS:

You were probably given one or more prescriptions for medications. Take all as directed on the bottle. Call us if you experience severe nausea or diarrhea, or cannot swallow your pills.

- **Antibiotics**: Continue until the bottle is empty. Do not quit halfway.
- **Pain Medicine**: Usually it is necessary to take narcotic pain medication in a scheduled fashion (every 4–6 hours) for the first 24 hours and as needed thereafter. Remember that narcotics can make you drowsy, so no driving, swimming, operating machinery, or drinking alcoholic beverages while you are taking them.
- You may wean yourself off of the narcotic medication and substitute 500 mg of acetaminophen (Tylenol®).
- If directed to do so, you may use ibuprofen, 600 mg, every 6 hours while awake, for the first 4 or 5 days.

ACTIVITY:

Once you are no longer taking narcotic pain medication, you may resume your normal activities as you feel up to it. Go easy at first with exercise and recreation, and increase your activity slowly over several days back to your normal routine.

Please follow these instructions as closely as possible. They are designed to help you heal quickly and comfortably.

QUESTIONS?

We are here for you.

If you have a question about your procedure or treatment plan, our center has 24-hour surgeon availability. If you would like to speak to your surgeon, please contact us at any time.

During business hours, please call our office. After hours, please call our office, and our answering service will page a surgeon. We'll get back to you as soon as possible.

Your usual general dental checkup involves **dental scaling** to remove tartar buildup. In the first few days, you might experience tooth sensitivity. All you need to do is follow post-care instructions to relieve any feeling of discomfort.
Your dentist conducts dental scaling and root planing to clean your teeth and gums. The combined procedure serves as a preventive gum disease treatment.
Dental scaling aims to fight off any inflammation that can lead to periodontal disease. Periodontal or gum disease is a common concern that worsens if left untreated.

Does dental scaling cause hypersensitivity?

It is essential for you to undergo teeth scaling as a part of a deep cleaning teeth procedure. Your dentist uses scaling tools to remove plaque and tartar from your teeth and gum line.
Since the scaling tool can go below the gum line, root planing exposes the tooth root briefly. During the first 24 hours, it is normal to experience tooth sensitivity.

Signs that may lead to tooth sensitivity

The common signs that can trigger sensitivity are as follows:

- Swelling

- Redness
- Inflammation

All these signs are normal. You'll need to wait for a few days before your gums can completely reattach to the teeth. You don't have to fret, as the discomfort you may be experiencing will soon pass.

Helpful reminders to relieve discomfort

For faster healing, you need to follow your dentist's aftercare instructions. Here are some tips you can follow to observe oral care at home.

What you need to do

- Stick to gentle brushing and flossing a few days after your dental cleaning. Once fully recovered, you can do the usual routine you're doing for your oral care.
- Gargle with a warm saltwater solution at least three times a day. You can create one by mixing 1 teaspoon of salt into 8 ounces of water.
- Rinse with a **fluoride solution**. Many over-the-counter types of mouthwash are available.
- Take the medications your dentist prescribed if there is any. If there is none, simply pop a pain reliever such as ibuprofen or acetaminophen every 6-8 hours.

What you need to avoid

- Eating hot foods like soup and drinking coffee and tea fresh from the pot can burn your mouth. At the same time, ice-cold drinks can trigger hypersensitivity.
- Eating spicy foods can irritate your gums and worsen the inflammation.
- Smoking in the first few days can delay your recovery period. Heat can expose your gum tissues, which can lead to prolonged healing.
- Consuming alcoholic beverages within 48 hours of any periodontal treatment can interfere with your healing process.
- Exercising or engaging in any strenuous physical activity can increase blood pressure. It dissolves the clot needed to hasten your recovery.

You may feel sensitivity during the first two days or so after your **dental scaling** appointment.

POST-OPERATIVE INSTRUCTIONS FOR TEMPORARY DENTAL CROWNS AND DENTAL BRIDGES

Please follow these instructions while you are awaiting your permanent crown or bridge. Do not hesitate to call the office should you have any questions about your temporary.

1. Avoid eating sticky foods (caramel, chewing gum, hard candy) on the side of the mouth with a temporary crown.
2. Do not floss the area around your temporary crown. Should you find it necessary to do so, pop your floss in between your teeth and then pull it out on the side. Do not pop the floss back out as it can cause the loss and/or damage of your temporary crown.

3. Your temporary plays an important role in protecting your tooth and holding the space for your permanent crown or bridge. Should it need recementing or become damaged please call our office during business hours and we will be happy to recement or repair it.
4. Should your temporary become uncemented on the weekend or while you are out of town, purchase **temporary dental cement** from a drugstore or use the packet attached here. Remove any cement left in the temporary and replace it with a “teardrop” amount of cement. Do not leave any cement on your gums.
5. It is normal to have significant changes in response to temperature and soreness at the injection site after dental restorations. This should subside within a few days and in some cases weeks. If your pain gets progressively worse or is causing more than mild discomfort, please call this office.
6. Ibuprofen (Advil, Motrin) is very effective for dental pain. Two to four tablets may be taken four times a day for the next 3-4 days (if needed) to help control the sensitivity in this area.
7. Should the gums around crown temporary crown become sensitive, rinse 4 times a day with a warm salt water solution (½ tsp salt to ½ cup water).

POST-OPERATIVE INSTRUCTIONS FOR DENTAL CROWNS

When you receive a dental crown, you are making an investment in your mouth. Please take the following steps to ensure that your dental crown will last you for years to come.

Brushing

- Brush twice a day for at least 2 minutes.
- It is possible to get a cavity under your crown. Pay special attention to your gumline. Plaque and bacteria at your gumline will lead to decay that can cause your crown to fail.
- A mechanical or ultrasonic brush, such as Sonicare, will help you to adequately remove plaque and bacteria. In addition, most electric brushes have a timer that assists you in brushing for a full 2 minutes.
- Pay special attention to the area where the crown meets your natural tooth and gums on the tongue side and cheek side. This area can trap bacteria and plaque which can lead to a cavity under your dental crown.

Flossing

- Floss all of your teeth at least once a day.
- Make sure that you insert your floss in between each tooth. You should hear a “pop.”
- Wrap the floss around each tooth and move it up and down to loosen and remove food and bacteria.
- Use a new section of floss for each tooth.
- Floss fingers and other appliances available at the grocery store can help you reach your back teeth easily.
- Waterpicks and Sonicare AirFloss are useful to help keep the bottom area of the open space between your teeth clean. You must still floss the contact where your dental crown and adjacent teeth meet (the “pop” area described above).

Fluoride

- Prescription strength fluoride can prevent new cavities and reduce gum inflammation caused by bacteria.

- Fluoride also reduces tooth sensitivity and soothes discomfort after tooth whitening.
- Fluoride is available in gel, rinse, and toothpaste forms as well as multiple flavors and strengths. Nicole, our hygienist, and Dr. Morgan can help you choose the fluoride that is best for you.

Dental Visits

- You should visit our office at least twice a year for professional dental cleanings or more often if directed.

POST-OPERATIVE INSTRUCTIONS FOR VENEERS

When you receive veneers, you are making an investment in your mouth. Please take the following steps to ensure that your veneers will last you for years to come.

Brushing

- Brush twice a day for at least 2 minutes.
- It is possible to get a cavity under your veneer. Pay special attention to your gumline. Plaque and bacteria at your gumline will lead to decay that can cause your veneer to fail.
- A mechanical or ultrasonic brush, such as Sonicare, will help you to adequately remove plaque and bacteria. In addition, most electric brushes have a timer that assists you in brushing for a full 2 minutes.
- Pay special attention to the area where your veneer meets your gums. This area can trap bacteria and plaque which can lead to a cavity under your veneer.

Flossing

- Floss all of your teeth at least once a day.
- Make sure that you insert your floss in between each tooth. You should hear a “pop.”
- Wrap the floss around each tooth and move it up and down to loosen and remove food and bacteria.
- Use a new section of floss for each tooth.
- Floss fingers and other appliances available at the grocery store can help you reach your back teeth easily.
- Waterpicks and AirFloss (by Sonicare) devices are useful to help keep the bottom area of the open space between your teeth clean. You must still floss the contact where your dental crown and adjacent teeth meet (the “pop” area described above).
-

Fluoride

- Prescription strength fluoride can prevent new cavities and reduce gum inflammation caused by bacteria.
- Fluoride also reduces tooth sensitivity and soothes discomfort after tooth whitening.
- Fluoride is available in gel, rinse, and toothpaste forms as well as multiple flavors and strengths. Our dental hygienist and Dr. Morgan can help you choose the fluoride that is best for you.

Dental Visits

- You should visit our office at least twice a year for professional dental cleanings or more often if directed.

- At least once per year x-rays will be taken and each tooth will be checked for cavities, cracks, and infection by Dr. Morgan. Please call our office at any time if you are experiencing any tooth problem or wish to see our hygienist or Dr. Morgan. We are here to help.

Whitening

- In order to keep your natural teeth the same color as your new veneers, periodic whitening is recommended.
- The most economical way to keep your smile white is to use custom whitening trays with a strong whitening solution. Daywhite whitening syringes or other whitening products can be purchased for a minimal price.
- Most patients find that the best time to whiten is after a professional dental cleaning. Your teeth are plaque and tartar free thus allowing your teeth to respond positively to the whitening gel. Let your teeth be your guide on how often you should whiten.

Occlusal Guards

- Clinching and grinding exerts pressure that can be generated across the teeth that can range from 100 to 600 psi (pounds per square inch). That incredible amount of force can cause many different problems related to your gums, jaw, and teeth.
- Clinching and grinding when you have multiple porcelain restorations can cause cracks and fractures to your natural teeth. Porcelain by nature is a harder material than natural enamel.
- An occlusal guard is an appliance designed from dental models of your teeth and made of a rigid plastic. It covers either your upper or lower teeth and prevents the teeth from coming together while sleeping at night. It also provides a guide for your jaw so that muscles can relax and bite problems will not trigger the bruxing action.

POST OP INSTRUCTIONS FOR CROWNS AND BRIDGES

First visit for Crown or Bridge work and temporary crown/bridge placement: During your visit today, one or more of your teeth was prepared for a crown or bridge. Due to the anesthetic you are numb and may be for a few hours (up to five hours). Please, be careful chewing on that side of your mouth or both sides (if both sides of your jaw were numbed today), so that you don’t bite your lip, tongue, or cheek. You have a temporary crown in place. Temporary crowns are cemented with a temporary cement, to be easier to remove at your next appointment. Please, be careful chewing, avoid anything hard, especially sticky food on that side, as that might break or force out the temporary crown, before your next visit. If your temporary crown falls off, please keep the piece and contact us as soon as possible, for re-cementation at no charge. If your temp falls out and you don’t come back to have a new one placed, there is a chance that the tooth might fracture and will result in complications such as not being able to restore the tooth with a crown. After a crown preparation, some discomfort is normal, especially if you also got a crown lengthening procedure done. During the procedure there is usually some disturbance to the nearby tissue. It is normal for gum around the tooth to feel sore or sensitive. Usually this will heal on its own; we advise to rinse with warm salt water around the affected area. Watch out for any signs of severe pain, throbbing on the tooth, difficulty sleeping lasting for days, as this may be a sign that the tooth is infected. Second visit for Crown or Bridge work and temporary crown/bridge placement: During your visit today your permanent crown/bridge will be delivered. Permanent crowns/ bridge are cemented with permanent cement. It is very important that you agree with the feel and shade of your crown before it is cemented. After the permanent crown is cemented, it is advised to avoid sticky things for the first 24 hours. After that, you may eat, drink, and clean your tooth just as you do

regularly. If your bite feels uneven please contact us as soon as possible, because high spots may hurt or damage the treated tooth.

Post operative instructions for composite restorations:

1. Sensitivity is usually most noticeable the first 12-24 hours after the anesthetic wears off. 2. Sensitivity, especially to cold, is common for a few days following a dental filling. Usually the deeper the cavity, the more sensitive the tooth may be. 3. The gum tissue could have been irritated during the procedure and may be sore for a few days together with the anesthetic injection site. 4. The filling is fully set and ready to eat on when you leave the office. Please be careful eating until the anesthetic wears off so you don't bite your lips, cheeks, or tongue. 5. Children should be observed until the anesthetic wears off. Due to the strange feeling of the anesthetic, many children will chew the inside of their lips, cheeks, or tongue which can cause serious damage. 6. The finished restoration may be contoured slightly different and have a different texture than the original tooth. Your tongue usually magnifies this small difference, but you will become accustomed to this in a few days. 7. If after a week or two, your teeth feel they do not touch correctly please call the office. This problem can be solved with a quick adjustment to the filling. 8. Fillings do not last forever. Like a new set of tires, fillings can wear and breakdown. Proper brushing and flossing is recommended to help you retain your fillings Having your teeth cleaned every six months and an exam and xrays every year will help prolong the life of your fillings.

Composite filling

- Once the feeling has returned, you may chew on your new filling as it is as hard as it is going to get.
- Test your bite to see if it feels balanced all over – sliding your teeth back and forth, and forward and backward (just like in the office near the end of the appointment).
- If it feels like you are touching your new filling first or you just can't get all of your teeth together comfortably, call the office for advisement or an appointment.
- It is common to experience sensitivity to hot and cold for a few weeks following a white filling restoration. The deeper the cavity removed, the more sensitivity experienced.
- It is recommended that you chew slowly and on the other side of your mouth if you experience sensitivity.
- If it has not subsided within 10 – 14 days, OR it gradually gets worse, call the office for advisement or an appointment.
- The gum tissue surrounding the filling and tooth, your jaw joint, as well as the area where the anesthetic was placed, can be irritated and sore for a few days. This is normal and will subside in 2 – 5 days.

- If you are able to take ibuprofen, take two tablets every 6-8 hours for two days to alleviate the inflammation and reduce the soreness.
- The finished restoration may be contoured a bit differently and have a different texture than what you were used too with your tooth. Your tongue will become accustomed to the new filling in a few days.

Composite/Amalgam Post-Operative Instructions

• Do not bite together hard or eat on fresh amalgam fillings for 2 to 3 hours. Composite fillings set up hard right away. • Children should be observed until the anesthetic wears off. Due to the strange feeling of the anesthetic, many children may chew the inside of their lip, cheeks or tongue and cause serious damage. • Sensitivity, especially to cold, is common for a few days following a dental restoration. Usually, the deeper the cavity, the more sensitive the tooth will be. • Sensitivity is usually most noticeable the first 12 to 24 hours after the anesthetic wears off. • The gum tissue could have been irritated during the procedure and may be sore for a few days together with the anesthetic injection site. • The finished restoration may be contoured slightly different and have a texture different than the original tooth. Your tongue usually magnifies this small difference, but you should become accustomed to this in a few days. If the bite seems high or doesn't feel right please call our office

Glass Ionomer Cement Post-Op Instructions

- Your child has had their teeth treated with a glass ionomer cement filling or sealant material today.
- For 48 hours, please eat soft foods only, and use caution to avoid hard, crunchy foods while the material reaches its maximum strength and hardness.

  Suggested foods:

Soup	Yogurt
Macaroni and cheese	Apple sauce
Scrambled eggs	Oatmeal
Smoothies	Mashed Potatoes
Ice cream	Jello

- It is always a good idea to avoid chewing ice or hard candies to prolong the life of your child's teeth and restorations.

<u>Glass Ionomer Cement (GIC) Post-Op Instructions</u>

- Your child has had their teeth treated with a glass ionomer cement filling or sealant material today.

- For 48 hours, please eat soft foods only, and use caution to avoid hard, crunchy foods while the material reaches its maximum strength and hardness.

  Suggested foods:

Soup	Yogurt
Macaroni and cheese	Apple sauce
Scrambled eggs	Oatmeal
Smoothies	Mashed Potatoes
Ice cream	Jello

- It is always a good idea to avoid chewing ice or hard candies to prolong the life of your child's teeth and restorations.

The First 24-48 Hours

Directly following periodontal or dental implant surgery, you may experience some minor bleeding, oozing, swelling, and/or bruising. This is normal.
To help minimize discomfort…

Do not:

- Drink through a straw, spit, or vigorously rinse your mouth
- Eat hot, spicy, crunchy, or citrusy foods
- Exercise
- Smoke or drink alcohol
- Floss or use a toothbrush on the affected area. Do not use an electric toothbrush
- Touch the surgical area with your fingers or tongue
- Pull or tug your lip to look at the surgical area

Instead:

- Drink liquids or eat foods at room temperature
- Apply light pressure to your gums with gauze, a tea bag, or clean cloth (to address any bleeding)
- Apply ice to the outside of your face to reduce swelling or discomfort — 20 minutes on, 10 minutes off for the first 24 hours

- After 24 hours, you may use warm, moist heat on your face for swelling as needed

The post-operative instructions after periodontal surgery listed below should be followed accurately in order to speed your recovery.

1. You may continue to brush and floss the untreated teeth as usual. However, do not vigorously rinse or spit within the first 24 hours. Beginning tomorrow rinse with the prescription mouthwash twice daily until the bottle is empty. You may also rinse with warm salt water (one-half teaspoon salt to one cup water).
2. Some discomfort is to be expected and is usually most severe during the first six to eight hours following surgery. Many patients are able to manage the discomfort with Ibuprofen (Advil) 800mg every six hours. If you have been given a prescription pain reliever, take with food as directed.
3. Swelling is to be expected and usually reaches its maximum during the first day or two. Apply ice to the side of the face 15 minutes on and 15 minutes off as often as possible for the first 48 hours. Frozen vegetable bags wrapped in dishtowels work nicely as they contour to the jaw.
4. Keep your head elevated at all times for the first five to seven days following surgery. This will help decrease swelling and pain.
5. The surgical site may continue to bleed slightly for a day or two. This is no cause for alarm. If heavy bleeding occurs, fold a piece of gauze (or use a wet or dry tea bag) and place over the bleeding area. Maintain firm pressure for 30 minutes. Avoid frequent pack changes, rinsing, and spitting or physical exertion until the bleeding has ceased.
6. A periodontal dressing is often placed around the teeth to protect the surgical area and should not be disturbed. A soft diet will help prevent dislodgement of the dressing. Do not chew in the surgical areas or where dressing is placed. Often times, only one side of the mouth has been treated and you can cut food into small pieces and chew on the opposite side. If small pieces are lost, and you have no discomfort, there is no reason for concern. If large pieces break off or if the entire dressing becomes loose in the first two days, please contact our office.
7. You may eat or drink what you feel up to once the local anesthetic has worn off. Avoid very hot foods or liquids for 24 hours. Soft foods like eggs, pastas, warm soups and yogurt are recommended. Drink plenty of fluids, but do not drink through a straw or create a sucking action in your mouth.
8. DO NOT SMOKE. Smoking will dramatically delay the healing process.
9. Keep lips moist with ChapStick or Vaseline to prevent chapping.
10. If you have been prescribed an antibiotic and are currently taking oral contraceptives you should use an alternate method of birth control for the remainder of this cycle.

Onlay-Inlay Post Op

Instructions Following Delivery of Your Onlay

1. The cement is already set and your restoration(s) is ready for immediate use.
2. You may experience some minor discomfort for the first few days following your treatment. This should diminish gradually over time.
3. If you have been given an anesthetic, this should wear off in a few hours. Please be careful not to bite your cheek or tongue. Also, do not eat or drink anything extremely hot or cold until the numbness has completely worn off.
4. The bite should feel normal when the anesthetic wears off. If it doesn't, it NEEDS to be adjusted. Please call and we will schedule a short appointment. If sensitivity to hot, cold or biting pressure persists longer than one week, please call our office for a follow-up evaluation.
5. The tooth will look natural in color, so you may not be able to notice the restoration.
6. Do not chew any extremely hard or crunchy foods with your new restoration. (Ice, hard candy, etc.) Also, do not bite anything with your teeth that can damage them such as fingernails, paper clips, pens, etc.). Anything that can damage your natural tooth, can damage your restoration.
7. We recommend professional maintenance and evaluation at least twice a year unless otherwise advised. If you have had a periodontal (gum disease) problem in the past, you should see us up to four times a year.
8. Proper care includes BRUSHING and FLOSSING daily. Proper maintenance will not only prolong the life of the restoration but it will also help to prevent problems elsewhere in your mouth.

Nitrous Oxide Post Op

Post-Treatment Instructions

1. Continue to watch your child for at least 6 hours after the appointment.
2. Do not allow your child to play on stairs, ride a bicycle, swim or bathe, or engage in any activity that requires balance for at least 6 hours after the appointment
3. Give lots of liquids as soon as possible after the appointment, and continue liquids for the remainder of the day.
4. Give Children’s Motrin or Children’s Tylenonl, as recommended by the manufacturer or by your pediatrician, if you child is uncomfortable
5. If you child has had any teeth removed, pleaser refer to your copy of written instructions, “Instructions following Oral Surgery”, if you have questions.

It is very common for children to experience elevated temperatures following treatment. Be assured that you child does not have an infection. The elevated temperature is the way a child's body reacts to physical stress. Be sure your child drinks plenty of fluids and use Children's Motrin or Children's Tylenol, as recommended by the manufacturer or by your pediatrician, as needed.

Should you have any questions or concerns, please do not hesitate to call the office.

The above information has been reviewed and explained to me, all of my questions have been answered, and I have been given a copy of these instructions.

Crown Post Op

Instructions Following Delivery of Your Crown

1. The cement is already set and your restoration(s) is ready for immediate use.
2. You may experience some minor discomfort for the first few days following your treatment. This should diminish gradually over time.
3. If you have been given an anesthetic, this should wear off in a few hours. Please be careful not to bite your cheek or tongue. Also, do not eat or drink anything extremely hot or cold until the numbness has completely worn off.
4. The bite should feel normal when the anesthetic wears off. If it doesn't, it NEEDS to be adjusted. Please call and we will schedule a short appointment. If sensitivity to hot, cold or biting pressure persists longer than one week, please call our office for a follow-up evaluation.
5. The tooth will look natural in color, so you may not be able to notice the restoration.
6. Do not chew any extremely hard or crunchy foods with your new restoration. (Ice, hard candy, etc.) Also, do not bite anything with your teeth that can damage them such as fingernails, paper clips, pens, etc.). Anything that can damage your natural tooth, can damage your restoration.
7. We recommend professional maintenance and evaluation at least twice a year unless otherwise advised by Dr.Blake and his team. If you have had a periodontal (gum disease) problem in the past, you should see us up to four times a year.
8. Proper care includes BRUSHING and FLOSSING daily. Proper maintenance will not only prolong the life of the restoration but it will also help to prevent problems elsewhere in your mouth.

Implant Post-Operative Instructions

Instructions following Implant procedures

A dental implant has been placed in your mouth. The implant may be located above or below the tissue. This type of implant has been selected for your situation because of the bone available to place an implant. The implant will usually take a period of three to six months to heal; depending upon your body's healing properties and the type of implant surgery.

Usually you are able to wear your present partial, denture, or flipper, if applicable. Sometimes it is necessary to leave it in the night of the surgery. It is important to keep the appliance as clean as possible during the healing period. Please ask the doctor if you have any questions.

Every consideration must be given to keep the surgical site clean and free of food particles.

NO SMOKING! Smoking is to be avoided for the time specified by the doctor. Smoking increases the heat in the surgical site and significantly lowers the body's ability to heal the site.

AVOID these after surgery: alcohol with post-operative medications, commercial mouth rinses and very hot fluids.

Gentle rinsing of the mouth should be started the day after surgery. Frequent gentle rinsing with lukewarm salt water will aid the healing process (add one half teaspoon of salt to a 6oz glass of water). You may also be provided with a chlorhexidine rinse (Perioguard). Rinse gently with this 3-4 times per day the first week after surgery but do NOT swish or gargle. Avoid the use of a water-pik tooth brush.

Bleeding: A small amount of bleeding following surgery should not alarm you. If the bleeding is excessive or continuous, please call us at once. Vigorous rinsing of the mouth prolongs bleeding by removing the clotting blood, so when rinsing your mouth, do it gently.

Diet: Following surgery it is best to restrict your diet to fluids and soft foods for the first day. Normal diet may then be resumed the following day, but you will want to avoid chewing on the implant site until the tissue is completely healed. Soft foods such as Jell-O, pudding, mashed potatoes, scrambled eggs and soups are suggested. If you have difficulty chewing, try blenderized foods or diet supplements such as Carnation Instant breakfast and Ensure.

For Women Only: Do not breast feed for 12 hours if you were sedated for surgery. If you are using oral contraceptives, please note that antibiotics and other medication may interfere with their effectiveness. An alternative form of birth control should be used for one complete cycle of birth control pills after the course of antibiotics or other medication is complete

Full Denture Patients: For the first few months following surgery, you will need to be on a soft diet where the implants were placed. Foods such as pudding, applesauce, scrambled eggs, soft flaky fish, pasta and casseroles are recommended. The soft diet can last as long as 2-6 months. Your surgeon will advise you regarding how long you need to continue the soft diet.

IV Site: If medication was given intravenously, you may have some tenderness or bruising around the site of injection. Moist heat (warm, we cloth) or dry heat (heating pad on low heat) to the area the next day will help to ease any discomfort. If IV sedation was used for your procedure, you cannot drive a car for the remainder of the day.

NO drinking through straws or any other item creating suction within the mouth. The use of a straw creates negative pressure in your mouth and will tend to loosen the sutures and removes clotting.

Numbness: Due to the local anesthetic, there may be numbness in the surgery site that may last 6-8 hours.

Pain: A certain amount of pain must be expected with all types of surgery. An appropriate pain medication has been prescribed for you. Please take it according to the directions. It is advised that you do not drive while taking the pain prescription. However, if only Ibuprofen or Tylenol is needed, most people can drive without a problem.

Partial Denture Patients: Chew foods only in the areas where you have natural teeth remaining. Do not chew hard food in the area of your implants for as long as 2-6 months. Your surgeon will advise you regarding how long you need to continue the soft diet.

Post-Operative Implant Hygiene: The first year following placement of your implant, oral hygiene maintenance will be required every 3 months to 6 months as directed by your dentist.

Swelling: Some swelling and minimal bruising is possible and is to be expected. IT IS NOT UNUSUAL. In most cases, swelling can be prevented/controlled. Apply the ice pack that has been given to you for a period of 20 minutes on and 20 minutes off during the day for the next two days. The application of ice to the outside of the face over the surgical area will minimize swelling. If after five days you still have unusual swelling or pain, please call the office.

Stiches: The stiches used will dissolve in 7-10 days following surgery. It is not unusual for small pieces of sutures to come out prior to this time. If the sutures need to be removed, you will be notified, and an appointment will be made for you. Avoid playing with your implant or caps with your tongue.

Implant Follow-Up: We will see you for the periodic check-ups until the implant has healed

nstructions Following Oral Surgery

When Should You Call The Office?

1. If profuse Bleeding continues after 3-4 hours of applied pressure(see “Bleeding” below)
2. If you are unable to maintain a nutritious diet after 48 hours.
3. If the pain and/or swelling increases after the third day.
4. If you have an allergic reaction to medications such as:

- skin rash
- elevated temperature
- hives
- nausea/vomiting
- dizziness/fainting
- blurred vision
- increased heart rate
- irregular heart beat

General Instructions

CONTINUE biting on the gauze pack placed by the nurse for 30 minutes then discard.
IF YOU ARE STILL BLEEDING after 30 minutes, replace with fresh gauze for another 30 minutes; Thereafter replace gauze every 20 minutes until bleeding has stopped.
DRINK plenty of liquids.
DO NOT chew on the gauze.
DO NOT rinse, spit or drink with a straw for 48 hours.
DO NOT eat hard, sharp, hot or spicy foods for 48 hours.
DO NOT drink carbonated or alcoholic beverages for 48 hours.
DO NOT exercise or do heavy lifting for 3 to 5 days after the surgery.
DO NOT smoke for 72 hours.
KEEP your appointment for follow-up so the doctor can check to see if you are healing properly.

What To Expect After Extractions

BLEEDING – Minor seepage is normal the first 24 hours. To slow or stop bleeding, bite with light pressure on a gauze pack placed over the surgical area. Pressure should be applied in 30 minute intervals during the first hour and 20 minute intervals thereafter repeated until the bleeding is under control. If bleeding persists after 4 hours, wet a tea bag and wrap in gauze and bite on it without changing for 2 hours. IT IS VERY IMPORTANT to wait to complete this step. Exercise and heavy lifting will raise your blood pressure and will dislodge the blood clot and bleeding will resume.

SWELLING – To prevent and/or minimize swelling, when instructed, apply ice packs at 10 minute intervals to the surgical area. After 72 hours, apply warm compresses to the area to relieve swelling. Swelling is a natural part of the healing process and can be expected for 3 days to several weeks depending on the nature and extent of the surgery. Warm salt water rinses may be done 24-48 hrs after to help with healing.

Use 1 tsp of salt to 1 cup of warm water and roll around gently in mouth. Dispose in sink.

PAIN – Following any surgical procedure, there will be some degree of pain, which is determined by your pain threshold. A prescription for pain medication may be provided. However, in most cases, a non-narcotic pain regimen consisting of Acetaminophen (Tylenol TM) and ibuprofen

(Advil TM) is all that is needed. These two medications, taken together, are as effective as a narcotic, but without the side effects of narcotics. If a narcotic has been prescribed, follow the directions carefully. If you have any questions about medication interactions, please call our office, your physician or pharmacist.

DISCOLORATION - Bruising after surgery is not unusual, as it is a natural part of healing.

The process of placing an orthodontic appliance is non-surgical, but it does require special post-procedural instructions to minimize patient discomfort and protect each patient’s appliance from damage. Failure to follow these instructions can prolong treatment, make treatment more costly, or even result in a dental or orthodontic emergency.

Did you know…

that it is normal for orthodontic patients to experience some pain or discomfort following orthodontic treatment? Usually, soreness occurs in the muscles and teeth and can last for one to two days. During this time, many patients find difficulty eating – especially foods that are hard or crunchy. To relieve discomfort, it is recommended that patients temporarily consume a diet of soft foods or otherwise cut harder foods, like apples, into very small bites.

Frequently Asked Questions

Do I need to follow my orthodontist's post-op instructions?

Yes. Every directive is given for a reason, so it is imperative that you follow doctor's recommendations between orthodontic appointments. Also, never leave your orthodontist's office without the supplies necessary to facilitate post-op care. This may include acquiring orthodontic wax to protect your cheeks and gums from poking wires, or obtaining enough elastic bands and cleaning aids to last until your next orthodontic visit.

What types of post-op instructions will I need to follow?

Instructions vary from patient to patient and according to treatment. But if you have a fixed orthodontic appliance, you will probably have to follow special dietary guidelines to prevent damage to your braces or other orthodontic appliance. Most dietary restrictions include avoiding foods that are very hard or sticky, such as ice and chewy candies. If you do not have a fixed orthodontic appliance, your post-op instructions will include information about storing and cleansing your device.

Will my post-op instructions apply to every visit?

Possibly. There may be some visits where you are told to wear elastics or activate your expander until your next visit. It is important to never leave your orthodontist's office until you have a thorough understanding of your responsibilities at home. Continue to use proper brushing and flossing techniques, visit your dentist for periodic cleanings and examinations, and contact your orthodontist's office if you have any post-op questions.

WHAT TO EXPECT:

Every orthodontic patient will experience a period of adjustment when his or her appliances are initially placed. The first few days of wearing braces typically take the most getting use to and are the most challenging. During this time you may feel a general soreness in your mouth and your teeth may be tender to biting pressures for a few days. Depending on the type of braces you have been given, it may also take a week or two for the lips, cheek, and tongue to get accustom to the presence of the appliances. It is important that you keep in mind that these sensations will pass. To help you cope with this initial short-term discomfort your orthodontist will recommend a number of remedies and tips. These techniques can also be utilized during any brief "achy" episodes that may follow a future adjustment visit. If you are wearing conventional braces, a supply of orthodontic wax and instructions as to how to apply it to a wire or a brace that has become irritating, will be provided.

While wearing braces you will need to be more careful about what you are eating as well as more diligent in keeping your teeth and appliances clean. Eating certain foods (especially hard, sticky, crunchy, or tough foods) and certain oral habits (like chewing ice, biting pens, or nail biting) can be very damaging to braces. Risky behavior and frequent appliance breakage can significantly prolong your treatment and possibly compromise the result. Although, there may be some foods to avoid during treatment, there are still many delicious and satisfying things that you can eat. You must remember, the important thing is to maintain a healthy and well-balanced diet.

PARTS OF YOUR BRACES:

Here is a diagram of everything that will be in your mouth for the next couple of years. It is important to learn a few basics so that if something does break or come loose, you can tell us what it is. Some things can wait, and some can't. If you know exactly what it is that's broken or loose, we can tell you if you need to come in right away or not.

Spacers

Simply stated, spacers create space. Eventually, you will have a couple of bands around your back teeth, and right now, there is probably no room to put those bands. Spacers are little rubber bands that separate those back teeth from the rest of your teeth just enough to place a band around them. You may be getting spacers today, or you may be getting them next time you come in. They may be a little uncomfortable,

but taking ibuprofen or some kind of pain reliever either before the appointment or as soon as possible afterward should help.

Rubber Bands/Elastics

When and if the doctors tell you to wear your rubber bands, just wear them! Why? First, if you don't wear them, you are going to be "stuck" in braces for much longer than you would normally be. Second, the doctors are going to know! They can tell if you've been wearing them, no matter what you might try to tell him. Rubber bands are a GOOD SIGN! They mean you are getting closer to the end of your treatment, so don't stop now!

MAINTENANCE:

Keeping your teeth, gums, and orthodontic appliances clean over the course of care is of the utmost importance. Food and plaque are easily trapped in the tiny spaces between your braces as well as underneath removable appliances or aligners. In the absence of good oral hygiene your teeth can become decayed or permanently stained, and your gums can become irritated and inflamed. There is also a greater risk of developing a dental infection.

Brushing after every meal, and flossing at least once a day, is the best way to insure that your teeth and gums remain healthy throughout treatment. It is also recommended that you brush your teeth after eating snacks. However, if you don't have a toothbrush on hand at this time, you can clean your mouth by rinsing vigorously with water. You may consider adding other useful items like a floss threader, a small interdental toothbrush (proxabrush), or an oral irrigator (water pick) to assist you in keeping your appliances, as well as all of the small spaces around your braces, clean. Make sure to see your general dentist regularly for routine care and to have a thorough dental cleaning.

Beyond watching the foods that you eat and maintaining good oral hygiene, it is extremely important to wear your appliances, or any other orthodontic accessories, exactly as directed. Faithfully wearing your removable appliances, aligners, headgear, and/or elastics as instructed promotes efficient tooth movement and bite correction. You should also contact your orthodontist when an appliance breaks, or if a wire or any part of your braces is irritating your mouth.

KEEPING APPOINTMENTS

If you miss a lot of appointments, guess what? You will be in braces longer than you have to be! Also, if you miss appointments without calling us to let us know, you may end up getting charged for these. It is very important that the doctors see you on a regular scheduled basis. If not properly monitored, braces can cause problems that can possibly cause you to have braces longer than expected.

FOLLOWING INSTRUCTIONS

It is very important to do what the doctors and the staff tells you to do. Following their instructions will allow you to keep your treatment time on schedule. Not doing what you are asked to do will extend your treatment time. You will be in braces longer if you do not follow directions.

Your Braces and Food!

We know you are worried about this, but rest assured, you will not starve! It will be difficult to eat for a few days, but you will get used to your braces pretty quickly and be eating normally soon. Have some soft food on hand for the day you get your braces on. Things like soup, yogurt, pudding, mashed potatoes, macaroni & cheese, & scrambled eggs will help you get through those first few days.

Extra Tips

1. Take extra precaution when eating fruits with pits as you could accidentally bite into the pit. This would include fruits like peaches and plus.
2. Cut meat off of the bone to ensure you won’t accidentally bite into the bone.
3. Avoid chewing pencils, fingernails and ice.
4. Do not bite into anything and then pull away with your teeth. This could pull on your braces leading to bending or breaking them.

Next, there are things you MUST AVOID with braces!

These are:

*Gum (sugar-free or regular)	*Licorice	*Ice	*Hard taco shells
*Sugar Daddies	*Toffee	*Nuts	*French bread crust/ro[illegible]
*Tootsie rolls	*Caramels	*Bagels	*Corn on the cob

*Carbonated drinks<br>*Now & Laters<br>*Gummy bears<br>*Jolly ranchers	*Skittles<br>*Starbursts<br>*Taffy	*Chips<br>*Pizza crust<br>*Lemon juice	*Apples (unless alrea[illegible]<br>*Carrots (unless alrea[illegible]<br>*Hard chocolates

BE PATIENT

All treatment times that we have talked about are only estimates. Everyone is different, and everyone's teeth move at different rates. Your teeth will "look done" to you long before they really are. The part of the tooth that you see moves faster than the root, which you don't see. If you take the braces off before the root has moved, guess what? The tooth will move right back to where the root wants it. Remember that the doctors are trained to see many things about our teeth that the rest of us do not. They will not keep you in braces any longer than they have to, but at the same time, they don't want to let you out of them before you are totally done. They take your teeth seriously!

Finally, taking care to protect your braces from damage and your mouth from injury is extremely important. Wearing a mouthguard while participating in sports is a good way to safeguard your appliances and smile. You must wear a mouthguard that has been specially designed to fit over braces. Your doctors can guide you in selecting the best one.

Achieving the best orthodontic outcome depends on teamwork. Remember, a healthy and beautiful smile that will last a lifetime is well worth the effort.

Post Operative Instructions for Braces

At Dantkriti Dental Clinic in Gurgaon. We try our best to keep our patients happy during their orthodontic (Braces) treatment.

1. **Brush and Floss!**It is important to brush and floss daily and to maintain oral hygiene. To assure the best treatment outcome it is necessary to keep teeth and gums healthy and maintain the quality of braces.
2. **Keep Your Appointments!** missing a lot of appointments means You will be in braces longer than you have to be and treatment duration will be prolonged.
3. **Be Patient!** All treatment duration that we have talked about are only Estimates as everyone's teeth move at a different pace. Your teeth will "look done" and seems they are perfectly aligned to you long

before they really are. The visible crown part of the tooth moves faster than the root. If you ask to remove the braces before the root has moved, the chances of relapse are high as the tooth will shift back to its initial position where the root wants it.

4. **Retainers:** Sometimes after the treatment is done orthodontist recommends the patient to wear retainers which could be either removable or fixed for a certain duration to avoid relapse and allow teeth fibers to adjust according to their newposition.

DO’S and DONT’S IMMEDIATELY AFTER WISDOM TOOTH REMOVAL OR ORAL SURGERY

- There might be some difficulty in eating for a few days, but soon you will get adjusted to your braces.
- Initially have some soft food for the day you get your braces like soup, yogurt, curd rice, mashed potatoes, macaroni,etc.

THINGS YOU MUST AVOID WITH BRACES!

1. Chewing Gum of any kind of sticky, chewy, and hard food like nuts, éclairs, taco chips, and Outer edges of the pizza.
2. Non-diluted lemon juice, aerated drinks like Cola, Pepsi. These can dissolve away the enamel on the surface of your tooth.
3. Chewing on ice should be avoided
4. **Hygiene**: Brush your teeth, gums braces and wires thoroughly after each meal and before going to bed. We recommend that you carry around a travel toothbrush. Poor oral hygiene can result in puffy, bleeding gums and permanent white spots on teeth. Inflammation and bleeding gums will delay your treatment. An interproximal brush is the best way to clean around your braces and can be purchased from reception; a demonstration will be given at your hygiene appointment. Use this brush between your teeth at the gum line. We do reserve the right to suspend or delay treatment if your oral hygiene is poor.

5. Please keep your teeth and braces clean.
6. **Hard Food**: Do not eat hard food such as popcorn, ice, boiled sweets, hard crusts etc. Those foods can break the brackets. Cut up foods such as meats, apples, carrots etc before eating them. (If abuse is noted on multiple periodic treatment visits, a fee of £100 may be applied to replace brackets). Remember to eat sensibly when wearing a brace.
7. **Coloured Foods**: Some foods/drinks will stain the elastics on your braces, so try to avoid any foods/drinks with strong colourings for example tomato based foods, curries, coffee and tea, it's advisable to have these kinds of things perhaps the day before your next visit, as we can always change your stained elastics to new clear ones.
8. **Soreness**: After the braces are put on the teeth they may be sore, usually for the first 2-4 days. Ibuprofen or paracetamol may be taken to relieve this. If the soreness continues even after eating soft food, please phone for an appointment so any necessary adjustments can be made. If the inside of the lips or generally the soft tissue areas are sore, the wax provided can be used as a cushion over the braces until the lips become accustomed. Taking painkillers prior to your adjustment appointments can help minimise the discomfort.
9. **Jaw Joint**: There are some patients who will develop popping/clicking or other problems in their jaw joint during or

after orthodontic treatment. This is very rare. Usually, orthodontic treatment provides a positive effect on the jaw joint. You should understand that pre-existing joint conditions can manifest as a popping or clicking after orthodontic treatment but orthodontic treatment by itself has not been shown to cause popping/clicking of the jaw joints.

10. **Cleanings**: You will get three hygienist appointments for a professional clean and home care advice during your treatment. If you have an appointment for a cleaning scheduled, keep it! This is not required but highly encouraged.

11. **What if I get problems?** Problems with fixed braces are unusual. However, if anything happens that makes the brace very uncomfortable and cannot be controlled using the remedies recommended above, or you notice that it is broken, please call us and ask for an appointment. If a bracket or the wire starts to cause any discomfort, just apply the orthodontic wax as demonstrated.

12. **Smile Care Pack**: Contains a packet of interproximal brushes, Interspace Toothbrush, Soft/Ortho Toothbrush, Orthodontic wax and a bottle of Fluoriguard mouthwash. This pack is complementary.

turning □ You will turn______________________________ (to be noted at your issue appointment). It is a good idea to write it on a calendar, or put a reminder on your phone of when you have to turn. If you miss a day, do not try to catch up, leave that day and continue as normal. fitting □ When fitting your twin block, always make sure that you look into a mirror so that you can correctly position the plates. Once your plate is in the correct position, simply use your fingers to push against the plastic and the plate will click into place. removal □ When removing your twin block, always use your index fingers to pull downwards on the metal clips

located on the far back sides of the plates. To remove the lower plate use your thumbs and push up. This will help prevent damage to the more delicate wires at the front of the appliance. speech □ In the first few days your speech will be altered and you will create extra saliva. This will reduce with time and practice. Reading out loud for 5 to 10 minutes a day will help you get your speech back to normal again. cleaning your twin block □ Your twin block should be cleaned every day with a soft toothbrush and warm running water (not hot water). The best time to clean your twin block is when you brush your teeth, as you will be near a sink and the plates need to be removed anyway. Plate cleaning tablets such as Sterident or Retainer Brite may also be used occasionally, if the twin block develops an odour over time. what we give you today □ A key - please keep in a safe place at home. If you misplace it, please let us know as soon as possible. A baby toothbrush - to help clean your twin block with. A case - to keep your twin block in, when it's not in your mouth or is being cleaned. A sample of Retainer Brite - for you to use once every few months. Place tablet and twin block in a glass of water for 15 mins and then brush with the baby toothbrush to thoroughly clean it. A twin block consists of two removable orthodontic plates that fits both the upper and lower teeth. They may include springs to move individual teeth around, however the main aim is to ensure that the lower teeth are guided forward by the orthodontic appliances, so that the upper and lower teeth are positioned over one another, producing the overlap of the upper and lower teeth, which is naturally occurring. expanding your twin block □ To create expansion with your twin block, you will be required to use a key to adjust your plate on a weekly basis, according to your Orthodontist's instructions.

Once this is completed, the key should simply slide out and a new hole should be visible at the beginning of the yellow arrow. The twin block is now ready for the next time you need to expand. You may also find that a space opens in the middle of the upper plate, confirming a correct rotation has occurred. expanding your twin block □ Turn your expansion screw as specified by your Orthodontist. To help you remember, do the expansion on the same day each week. Turning is best done at night before bed, so that you get used to the expansion over night. You should feel some pressure after the plate has been expanded each time and in a few hours this pressure will subside. wear □ Unless otherwise instructed, your plates should be worn almost 24 hours a day. This means that you leave your twin block in place for sleeping and eating soft foods. The only times you may remove your plates is when you are cleaning your teeth, playing a contact sport, eating very hard or sticky food, or swimming at the beach. Note: not wearing your twin block will result in a poor fit, which can be uncomfortable and will prevent you from achieving the best result

Protraction Face Mask Why is this used? Some patients have a bite relationship in which the upper teeth are behind the lower teeth which is called an "underbite". This is caused by either the lower jaw growing excessively forward, the upper jaw not growing forward enough or combination of both. A Protraction Facemask is used to correct these "underbites". When is it used? It is used on actively growing patients. It is not effective on adults because their growth is complete. The appliance is worn a minimum 12-14 hours per day (after school and bed time). The more often it is worn; the less time it will take to work. How does this work? The upper pad rests on the forehead and the lower pad rests on the chin. Rubber bands (elastics) are placed on the pins and connect to the hooks on the appliance in the mouth. When stretched, the rubber bands will apply a light force to the upper jaw. This light force helps to move the upper jaw and upper teeth forward. How should I sleep? Most patients are able to sleep normally with the

appliance after a 2-4 day period of adjustment. Of course, every patient is different. It is most comfortable to sleep on your back or on your sides. Gentle encouragement at home is helpful. How soon will I see changes? The changes will be noticeable within the first month. Due to individual variation, every patient responds differently. Since the upper teeth are moving forward, there may be a period in which the upper and lower teeth contact on the edges. This is normal and will soon pass once the bite is completely corrected. How many months will it be worn? The amount of time is dependent on the severity of the under bite, the amount of appliance wear and jaw growth. The average patient will wear the appliance for 6-12 months. Once the bite is corrected, what happens next? Once the bite is corrected, minimal retention is needed because the lower teeth usually prevent the upper teeth from moving back to their original position. In severe cases, an early set of braces (Phase I treatment-partial braces) will be recommended braces are placed after full eruption of all of the teeth. Things to remember: It is very important to wear your facemask for the specified time and duration. Forgetting to wear your facemask will reduce the success, increase your treatment time, and in some cases make our goals impossible to achieve. Wearing your facemask may help prevent the future need of having permanent teeth removed and/or jaw surgery.

Post-Operative Instructions: After Jaw Fracture Surgery

Following these instructions and the instructions the nurses have given to you will result in fewer complications and make your recovery period easier. Failure to follow these instructions could result in unnecessary pain, delay in healing, or complications, which could negatively affect the outcome of your treatment.

Following surgery, your jaws may be held together with elastics. A period of 2–6 weeks is usually required for initial bone healing.

What You Need For Home

- Syringe
- Saline (to make your own: dissolve 1 teaspoon of salt in an 8-ounce glass of warm water)
- Child sized toothbrush/Waterpik®
- Scissors/pocket knife (for cutting elastics if your jaws are held closed)
- Vaseline or lip balm
- Blender or food processor

Medications/Prescriptions

If necessary, a prescription for medications will be provided at the time of your discharge. Please take the medication as prescribed until it is finished. You may be sent home with a prescription for a liquid pain reliever, which can be administered through a syringe as you have been shown,

or sipped from a spoon. If your pain reliever is in pill form, you can crush it and mix it with 10–20 ml of water or juice to be supped or administered through a syringe.

You may also be given a prescription for a liquid antibiotic to prevent infection. It is important to take this medication as prescribed until it is finished. You may also be given a prescription for an antibiotic mouth rinse. It is very important to keep your mouth clean.

An increase in swelling and pain after the first week could indicate an infection, which may require treatment. Should this happen to you, contact your doctor.

Care of The Operative Area

Swelling

For the first 48 hours after surgery, you will be given ice packs, which will help to minimize swelling. Following this period, you will need to use heat (hot, wet facecloth, hot water bottle, heating pad, or microwaveable pack) to help reduce the remaining bruising and swelling. As it takes about 2 weeks for the majority of the swelling to disappear, continue to use heat for 30–45 minutes, 4–5 times a day for at least 1–2 weeks after you are discharged from the hospital. A few minutes of gentle massage while using the heat also helps.

Bleeding

Prolonged bleeding, such as nosebleeds or bleeding from the incision sites following discharge from the hospital, is not normal, and you should contact your doctor if this occurs.

Sore Throat

For the first couple of days following surgery, you may experience a sore throat and some nasal decongestion. This is normal after anesthesia and should go away within a couple of days. Drinking plenty of liquids usually helps with the throat tenderness.

Lip Care

You will be unable to keep your lips moist when your jaws are held together with elastics. In addition, cracking of the corners of the mouth does sometimes occur following surgery. Apply Vaseline or lip balm regularly to keep these areas from becoming too dry or chapped.

Oral Hygiene/Mouth Care

It is important to remember to clean your teeth and rinse your mouth routinely following surgery. Using a Waterpik® after the first week is an excellent aid. A mild salt solution or a commercial mouthwash (non-alcohol based) will assist you in keeping your mouth clean. It is important to

rinse your mouth with 20–30 ml of saline frequently every 2 hours as well as after meals. You can make your own saline (see above). Use a child toothbrush to clean the outside of your teeth. You can start brushing the front of your teeth as soon as it is not too painful and progress to the back of your mouth when the swelling in your cheeks comes down. You must do this as thoroughly as possible. You will, of course, not be able to brush the tongue side of your teeth with a brush. The tongue side of the teeth can be brushed by moving your tongue across them while using a mouth rinse. You should avoid carbonated beverages, as they tend to decalcify your teeth.

Muscle Spasm and Mobilization

Occasionally, several elastics will break away during the fixation (teeth together) period. As long as you cannot open your mouth significantly, this is not a problem, and elastics will be replaced at one of your post-operative visits. If a large number of elastics are lost, and you can open your mouth, don't be alarmed. You should, however, contact your doctor so that new elastics can be placed.

Diet

Since your jaws may be held together with elastics, you will require what is called a balanced fluid diet (blenderized). It is essential that your body receives adequate fluids and nourishment in order to maintain your nutritional status and promote healing.

You will be limited to a strictly liquid diet until your jaw is no longer tightly held together. During this period, you will become creative with your menu choices. It is especially important to drink adequate amounts of fluids, 3–4 liters per day. You can purchase liquid nutritional supplements (such as Ensure or Boost) in a grocery store. You may continue to use the syringe for feeding, or when you are comfortable, use a straw or drink from a glass. A nutritious dietary intake is important to promote healing and decreasing the possibility of infection. You can expect to about 5–10% of your total body weight during the first 6 weeks following your surgery. A rapid loss of weight during the first week is usually due to fluid loss.

After the first 6 weeks, you can progress slowly to a normal diet. The first 4 weeks following the removal of the tight elastics, your diet should involve soft foods (eggs, potatoes, fish, pasta, etc.).

Here are some tips for creating a personal menu:

- You may eat anything that can be thinned into liquid form. Meals may be blenderized until smooth. If food is still lumpy, use a strainer.
- Cold whole milk can be used to thin puddings, yogurt, cereal, sandwiches, ice cream, and cakes.
- Warm whole milk can be used to thin cheese, eggs, toast, hot cereal, muffins, pasta, hot main dishes, and casseroles.
- Fruit juice can be used to thin fruit, yogurt, and ice cream.

Weight loss is a common result of a liquid diet. If you are experiencing weight loss, try snacking between meals and adding whole milk cheese or skim milk powder to meals to boost caloric intake. Constipation may result from the low fiber content in liquid diets or may be a side effect of some pain medications. To avoid this, try to include a lot of fruits and vegetables in your diet, and add prune juice to your daily menu.

Alcohol and smoking can delay wound healing and promote infection. Alcohol and smoking should be avoided until your surgical sites are completely healed.

Choking

In the unlikely possibility that choking or breathing difficulties may occur, we recommend that you have scissors or a pocket knife with you at all times while your teeth are wired together. In the rare event that you need to cut the elastics, proceed with cutting the elastics and then contact your doctor immediately. The nurses will instruction in the art of cutting the elastics in the event of an emergency.

Nausea

Avoid alcohol or foods that may cause your stomach to become upset. Should you experience nausea, you can use over-the-counter anti-nausea medication as directed on the bottle. If the nausea persists, please contact your doctor.

In most cases of vomiting, the elastics do not require removal. It is extremely rare to have to remove the elastics as the stomach contents are of liquid nature and can escape through and around the teeth. If emergency elastic removal (for vomiting or breathing difficulties) is required, please contact your doctor immediately. Remember that during the tight fixation period (with elastics), you should carry scissors or a pocketknife with you wherever you go.

Warning Signs of Complications

The following symptoms may be a sign of infection or other complications; therefore, you should follow up immediately with your doctor if they occur.

- Redness
- Increased swelling
- Increased or excessive pain
- Foul odor from the mouth
- Fever and/or chills
- Bleeding inside the mouth (wires may need to be adjusted)

Physical Activity

Physical activity should be kept to a minimum for at least 6–8 weeks after surgery. It is very important that you realize that you just had a significant operation that requires a well-rested recovery period. Excessive activity (running, exercising, swimming, heavy lifting, house cleaning, contact sports, going up and down stairs quickly, etc.) can cause bleeding and/or dizziness. If you had upper jaw fracture, you should avoid bending over during this time period as it may cause dizziness.

Excessive fatigue can also slow the healing process as well as increase the chance of infection by reducing your resistance. A gradual increase back to normal activity is the most sensible approach. Contact or other sports in which direct physical contact or injury are possible should be avoided for 2–3 months to minimize the risk of another fracture. If you have any specific activities you wish to perform following your surgery, please discuss this with your doctor.

Follow-Up With Your Doctor

A follow-up appointment should be arranged with your doctor's office prior to discharge. If an appointment has not been made, please call your doctor's office during regular business hours to arrange a follow-up appointment.

Please follow any other instructions that have been explained to you by your doctor.

Please review these tips for the safe use and proper disposal of prescription medications.

Following Surgery:

When you leave our office, your mouth will be numb and you will have gauze in your mouth for you to bite down on to control bleeding. Keep the gauze in place for one hour. After that, you can take it out and discard it.

- Don't touch the wound area or rinse your mouth vigorously.
- Avoid spitting, smoking or drinking from a straw as this can dislodge the formed blood clot causing bleeding or dry socket.
- Start taking your prescribed pain medication one hour after leaving the office with soft food.
- Restrict your activities on the day of surgery and resume them when you feel comfortable.
- Place ice packs on the sides of your face 30 minutes on and off.
- Keep your head elevated by sleeping in a recliner chair or propped up on a sofa for 72 hours.
- If you receive an irrigation syringe in your bag, do NOT use it for one week following surgery. After one week of healing, you may fill the syringe with mouthwash and water or warm salt water and irrigate the lower wisdom tooth

extraction sockets. You’ll do this after meals and when you feel food trapped in the healing sockets.

Jaw surgery

In the majority of patients, the process begins months before your surgery with orthodontic treatment. You’ll start by wearing braces to straighten your teeth. Pre-surgical orthodontics/braces typically lasts around 12 months but can be more or less depending on the severity of the jaw discrepancy. Your orthodontist will resume orthodontic tooth movements one to two months following surgery. Post-surgical orthodontics usually lasts about 12 months but can vary based on your specific needs. Your surgery will take place in the operating room as an outpatient procedure or with an overnight stay in the hospital. Be sure to follow all post-operative instructions carefully when you go home. Most patients are able to return to school or work in two to four weeks. It can take between four and six weeks for the bone to heal and harden so that you can chew properly. Stick with soft foods and liquids during this recuperation period and avoid exercising. It can take up to a year for full jaw function to be restored. You’ll come in for regular appointments with us for the first few months after your procedure. Once your jaw has started to heal, your orthodontist will continue orthodontic treatment with braces to lock the bite into place and fine-tune any details.

Following implant Surgery:

We’ll place gauze in your mouth before you leave our office. Biting down on it will help to keep bleeding under control. Leave it in place for an hour. After that time, you can take it out and throw it away.

- Don’t vigorously rinse your mouth or touch the wound area.
- Try to avoid touching the sutures with your tongue. This is easier said than done. Keeping gauze in place will help protect the sutures from early dislodgement.
- After dental implant surgery, you may have a metal or plastic healing abutment protruding through your gums. The healing abutment will shape and form the gum tissue while the bone is healing around the implant. After one week of healing, you may gently brush the abutment with a soft toothbrush.
- Don’t spit, smoke or drink from a straw.
- Begin taking your prescribed pain medication an hour after you leave our office.
- On the day of your surgery, restrict your activities. You can resume them when you feel comfortable unless otherwise instructed.
- During the first 72 hours, apply an ice pack to the side of your face for 30 minutes on and 30 minutes off.
- Keep your head elevated by sleeping propped up on the sofa or in a recliner for 72 hours.
- If you had a sinus lift, don’t blow your nose for two weeks following the procedure as it can disrupt the grafted bone and lead to graft failure. You should also avoid blowing your nose for two weeks after All-on-4 surgery.

Instructions after dental trauma

Activity

Please follow the instructions below:

- Keep teeth clean. Brush gently twice a day.
- If put in, keep dental splint clean. Brush gently twice a day.
- Do not bite directly on the injured teeth until the dentist says it is OK.
- Eat only **soft foods** for at least for 2 to 3 weeks. Examples include yogurt, mashed potatoes, soup, gelatin, and pudding. Avoid foods that are very hot or very cold.
- Do not play contact sports until the dentist says it is OK.
- Wait to get braces until the dentist says it is OK.

Medicines

Based on the type of dental injury, the dentist may recommend your child use a mouth rinse and take medicines, such as antibiotics.

For pain, your child can take over-the-counter medicine like ibuprofen (Motrin®) or acetaminophen (Tylenol®). Read the label on the bottle to know the right dose for the age of your child. Do not give aspirin to children. Never give them medicine for adults.

Follow Up Appointments

It is very important to keep all follow up appointments. Even if a tooth does not hurt after the injury, it still needs to be checked at specific times by the dentist. If injured teeth are not checked at these times, the teeth may be damaged forever or lost.

The length of time between follow-up visits to the dentist may vary from a few weeks to many years, depending on how bad the injury was. When a child receives timely care and proper treatment, the chance of tooth loss is reduced.

It is best to return to the Nationwide Children’s Hospital Dental Clinic or to the dentist who treated your child’s injury for all related follow-up appointments. If your child was seen in the dental clinic and you choose to follow-up with their own dentist, please ask the dentist to call the clinic to coordinate your child’s care.

When to Call the Dentist

Call the **Dental Clinic** if your child:

- has a fever over 102° F (38.9° C) and looks very sick
- a part of the face or around the tooth swells
- the tooth changes color or turns grey or black
- the gums around the tooth gets a bump, changes color or becomes sore
- the splint comes loose
- the temporary filling comes loose or falls out
- there is a lot of bleeding or pain at the injured site

Trauma Post Operative Instructions For Permanent Teeth Maintain soft diet for 2-3 weeks Examples: Mashed potatoes, Jello, Yogurt, Pudding, Applesauce, Grits, etc No biting into any hard or crunchy food with front teeth No Drinking through a straw Also limit spitting Sensitivity May need to limit very hot and very cold food or drinks Salt Water Rinses Recommended for at least 3 days Avoid Contact Sports To Keep Area Clean Use wet washcloth if not able to brush with toothbrush Tylenol Or Motrin Tylenol Or Motrin As need for pain

Trauma Post Operative Instructions For Primary Teeth Maintain soft diet for 2-3 weeks Examples: Mashed potatoes, Jello, Yogurt, Pudding, Applesauce, Grits, etc No biting into any hard or crunchy food with front teeth No Drinking through a straw or use of sippy cups Use a regular cup for all ages for the next 2-3 weeks Also limit spitting Sensitivity May need to limit very hot and very cold food or drinks Salt Water rinses recommended for at least 3 days If able to swish and spit Avoid Contact Sports What To Watch For At Home Tooth may turn a grayish color due to trauma to the tooth Also watch for swelling/bubble above the tooth, this may be a sign of an abscess. Please call our office immediately for evaluation If Tooth Is Loose It may take 1-2 weeks to tighten back up, avoiding testing to see if tooth has tightened back up because that could cause tooth to become looser. To Keep Area Clean Use wet washcloth if not able to brush with toothbrush

Trauma Post Operative Instructions For Patient With A Splint Take All Antibiotics If prescribed by the doctor Tetanus booster required if more than 5 years since booster If Splint Loosens Contact office immediately Salt Water rinses recommended for at least 3 days Or Chlorhexidine If prescribed Maintain soft diet for 2-3 weeks Examples: Mashed potatoes, Jello, Yogurt, Pudding, Applesauce, Grits, etc No biting into anything with front teeth Avoid Contact Sports (Including Band Instruments that Involves The Mouth)

Crown & Bridge Inlay & Onlay Instructions

How Long Will I Be Numb?

Your lips, teeth, cheek, and/or tongue may be numb for several hours after the procedure. To avoid injury, you should avoid any chewing or hot beverages until the numbness has completely worn off. It is very easy to bite or burn your tongue, cheek, or lip while you are numb.

Is It Normal for My Gums or Jaw to Be Sore?

Irritation can occur from dental work. Rinsing your mouth 2-3 times per day with ½ teaspoon of warm salt water can help to alleviate discomfort. Injection sites can be sore for several days after your appointment. Over-the-counter pain medications work well to alleviate the tenderness.

My Bite Feels Off – Is This Normal?

If your bite feels uneven, we can make a quick adjustment to the temporary or final restoration. Do not assume your bite will get better after time. The tooth may become irritated, and a toothache may occur if this is not done. It may also cause an increase in temperature sensitivity with the tooth.

Is It Normal for My Tooth to Be Sensitive?

It is normal to experience some sensitivity to heat, cold, sweets, and pressure after your appointment. It is common for this sensitivity to last several months after treatment. Usually, deeper cavities will be more sensitive. Again, make sure that your bite feels even.

Root Canal Instructions

How Long Will I Be Numb?

Your lips, teeth, cheek, and/or tongue may be numb for several hours after the procedure. To avoid injury, you should avoid any chewing or hot beverages until the numbness has completely worn off. It is very easy to bite or burn your tongue, cheek, or lip while you are numb.

Is It Normal for My Gums or Jaw to Be Sore?

Irritation to the gum tissue can occur from dental work. Rinsing your mouth 2-3 times per day with 1/2 teaspoon of warm salt water can help to alleviate discomfort. Injection sites can be sore several days after your appointment. Over-the-counter pain medications work well to alleviate the tenderness.

Is It Normal for My Tooth to Ache?

It is not uncommon for a tooth to feel tender or even exhibit a dull ache over the next few days as your body undergoes the natural healing process. These symptoms are temporary (usually lasting less than one week) and typically respond well to over-the-counter pain medications.

Why Does My Tooth Feel Different?

You may feel a slight indentation or rough area on the back of a "front tooth" or the top of a "back tooth". This is where a temporary material was placed through the access that was made on the tooth. It is not unusual for a thin layer to wear off in-between appointments. However, if you think the entire filling has come out, please contact our office.

Is It Normal for My Tooth to Be Sensitive?

Your tooth may be sensitive to biting pressure and may appear to feel loose. This is a normal response and is no cause for alarm.

My Bite Feels Off – Is This Normal?

Is It Okay to Eat on the Tooth with the Root Canal?

Please avoid chewing on the tooth that had the root canal. Whenever possible, try to chew on the opposite side of the treated tooth (as it is brittle) until the final restoration has been placed. The temporary filling will not permanently protect your tooth from reinfection or fracture.

Do I Need Any Other Treatment After a Root Canal?

A permanent restoration is required after a root canal is performed on a tooth. The location of the tooth will determine what restoration will be recommended. Most teeth will require some type of crown, although there are exceptions where a filling may be placed. This permanent restoration is used to avoid future tooth fractures as the tooth is “hollowed out” from the root canal procedure.

Extraction Instructions

Numbness

Your lips, teeth, cheek, and/or tongue may be numb for several hours after the procedure. To avoid injury, you should avoid any chewing or hot beverages until the numbness has completely worn off. It is very easy to bite or burn your tongue, cheek, or lip while you are numb.

Bleeding

Biting on a ***moistened*** gauze until the bleeding subsides is the best way to control the bleeding. Change the gauze as it becomes saturated with new ***moistened*** gauze. If bleeding persists after 2-3 hours, keep your head elevated and sit upright. You may try “home remedies” such as a moistened tea bag placed on the site for 30 minutes to help control bleeding. Overnight, it is normal to “ooze” from the area of surgery.

Bruising & Swelling

You may experience some bruising in the area of the surgery. In some people, this is a normal response and should resolve within 7-14 days after surgery. Swelling is best controlled by the immediate application of ice packs for 24 hours following surgery. Ice packs should be applied to the outside of the face in intervals of 20 minutes on and off.

Limited Opening

Jaw muscle stiffness with some limited opening of your mouth may occur after the removal of teeth. This is a normal response and may take several weeks to improve. Using warm, moistened towels on your face 24 hours ***after*** surgery may improve stiffness.

Fever

There may be a slight elevation of temperature for the first 24-48 hours after surgery. If a fever is present, it is extremely important to drink plenty of fluids

Eating and Drinking

Drinking plenty of fluids is essential. ***Do not suck through a straw***, as this will promote bleeding. Eating soft, nutritious food is encouraged as soon as the bleeding has stopped for the first few days after surgery.

Brushing & Rinsing

For the first 24 hours, do not brush or rinse your teeth as this may disrupt healing. After 24 hours, gentle brushing with a ***soft*** toothbrush is encouraged but avoid the extraction site. Avoid brushing sutures if placed. You may use a warm salt water rinse (½ teaspoon of salt mixed with 8 oz. of warm water) 3-4 times per day after the first 24 hours to help soothe the discomfort. ***Do not spit*** for 48 hours after surgery.

Smoking

Do not smoke for at least 24 hours after surgery. Smoking adversely affects oral hygiene and contributes to “dry socket”. Spitting and rinsing too early after surgery may also contribute to this painful condition.

Exercise

Please refrain from strenuous activity until 48 hours after surgery, as this will promote bleeding and interfere with the healing process.

Medications

You may be prescribed medications. Take them as directed to help control the discomfort. To avoid nausea, do not take pain medications on an empty stomach. ***Please note: some antibiotics may interfere with the effectiveness of your birth control pills.*** Please check with your pharmacist.

Implant Instructions

Numbness

Your lips, teeth, cheek, and/or tongue may be numb for several hours after the procedure. To avoid injury, you should avoid any chewing or hot beverages until the numbness has completely worn off. It is very easy to bite or burn your tongue, cheek, or lip while you are numb.

Bleeding

Some minor bleeding is expected after implant surgery. Upper implants may cause some bleeding from the nose. It should not alarm you and should subside quickly. Biting on a moistened gauze until the bleeding subsides is the best way to control bleeding. If a bone graft was placed, do not put pressure on the area. You may try “home remedies” such as a moistened tea bag placed on the site for 30 minutes to control the bleeding

Bruising and Swelling

Most patients will experience some swelling after surgery in the mouth. It is best controlled by the immediate application of ice for the first 24 hours following surgery. Ice packs should be applied to the outside of the face in intervals of 20 minutes on and 20 minutes off. Sleep with your head elevated slightly above the heart. This will help bring the swelling down. Some bruising may occur and should resolve within 7-14 days after surgery.

Eating and Drinking

Drinking plenty of fluids is essential. ***Do not suck through a straw*** as this will promote bleeding. Eating soft, nutritious food is encouraged as soon as the bleeding has stopped and for the first few days after surgery.

Fever

There may be a slight elevation of temperature for the first 24-48 hours after surgery. If a fever is present, it is extremely important to drink plenty of fluids

Brushing & Rinsing

For the first 24 hours, do not brush or rinse your teeth as this may disrupt healing. After 24 hours, gentle brushing with a ***soft*** toothbrush is encouraged but avoid the implant site and sutures if they were placed. You may use a warm salt water rinse (½ teaspoon of salt mixed with 8 oz. of warm water) 3-4 times per day after the first 24 hours to help soothe the discomfort. ***Do not spit*** for 48 hours after surgery.

Medications

You may be prescribed medications. Take them as directed to help control the discomfort. To avoid nausea, do not take pain medications on an empty stomach. Please note: some antibiotics may interfere with the effectiveness of your birth control pills. ***Please check with your pharmacist.***

These instructions are designed to promote healing, safety and comfort.**Fracture**

- Keep head elevated above your shoulders while sleeping for the first 3 days
- Use wax over sharp wires for comfort
- Use Vaseline or another soothing lip balm on lips as needed for comfort
- Excellent oral hygiene is MANDATORY. Gently brush teeth with small, soft brush and toothpaste, and rinse with warm salt water (1/2 tsp in 8 oz water)
- Proper nutrition must be maintained – high protein, high-calorie liquid diet.
- Do not drink alcohol or use recreational drugs.
- Do not smoke
- Do not use mouthwash or peroxide
- Avoid any further trauma to jaw. Restrict further exertion. Walking is encouraged.
- Notify us for loose wired, ability to move jaw, teeth, fever, increasing swelling, redness, pain or drainage

- Go to the Emergency Department immediately for difficulty in breathing or swallowing.

Take medications

Post-Operative Instructions at Oral Surgery Group

These are general instructions and not all of these instructions may apply to your recovery. Common sense will often dictate what you should do; however, when in doubt, follow these guidelines or call our office at any time for clarification.

The First Hour After Surgery

Bite down firmly but gently on the gauze packs that have been placed over the surgical area(s). Make sure that the gauze remains in place, undisturbed for 30 minutes, then reapply with clean gauze. You can also use a damp tea bag in place of the gauze. No eating, drinking, or sleeping with gauze in your mouth.

If you experience continued heavy bleeding while biting on the gauze, it means the gauze is in the wrong position and not pressing on the extraction site. Repeat the following steps:

1. Remove gauze.
2. Reposition gauze or tea bag so that pressure is applied to the bleeding site.
3. Bite on the gauze for 30 minutes to 1 hour.

Bleeding and Oozing

Intermittent oozing is normal for up to 24 hours and can last longer if you are taking blood thinners. The blood will mix with saliva, so it is important to determine if there is active bleeding. The above instructions can be repeated, and then the gauze can be discontinued when the bleeding has subsided. Do not go to sleep with any gauze in your mouth.

Pain

Unfortunately, most oral surgery procedures are accompanied by some degree of discomfort. There are many good strategies to get you through the process, though. Some form of pain reliever should be taken before the numbness goes away. Over-the-counter medications like ibuprofen (Advil® or Motrin®), acetaminophen (Tylenol®), or aspirin are adequate if there are no allergies, they have been tolerated in the past, and they do not interfere with any other medications. For more involved procedures, a prescribed narcotic can be taken. The narcotic can also be alternated or taken in addition to ibuprofen if there are no allergies and has been tolerated in the past. Taking your medications with food will decrease the chance of nausea. Remember, narcotic pain medications will impair your judgment and reflexes, so driving and operating heavy machinery is to be avoided.

Antibiotics

Antibiotics are prescribed to treat or prevent infections, so take all of the medication as directed. If you experience any adverse reactions, such as nausea, rash, or itching, discontinue the medication. A rash or itching may indicate an allergic reaction to a medication. Antihistamines (Benadryl®) will usually counteract the hives, rash, and itching. Swelling of the lips or tongue or difficulty breathing may represent a more severe allergic reaction, and you should seek medical attention immediately.

Swelling

Swelling from oral surgery is normal and reaches its maximum in 48 hours. Keeping the head elevated with 2 pillows when lying down can minimize facial swelling. Swelling can also be minimized by applying cold compresses to your face every hour (30 minutes on, 30 minutes off) for the first 48 hours. After 48 hours, warm compresses will help reduce the swelling. These should be continued (30 minutes on, 30 minutes off) until the swelling has subsided.

Rinsing

Avoid rinsing the mouth for 24 hours after surgery. It may disturb the clot. The following day, you may begin gently rinsing with warm (not hot) salt water. Dilute 1 teaspoon of salt in 8 ounces of warm water and rinse gently 3 or 4 times a day for 1 week.

Spitting

Do not spit because this action can disrupt the blood clot and/or promote bleeding.

Brushing

Avoid brushing near the surgical sites the day of surgery, since there may be stitches that can be disturbed, as well as some soreness and swelling. Begin your normal hygiene routine as soon as possible and gently brush the areas you are comfortable with.

Irrigation Syringe

If you have been given an irrigation syringe, start using 1 week after the procedure. Fill it with warm water and use gently to clean the socket. The socket will be closing up from the bottom and after 3 or 4 weeks will close up, and food will no longer get stuck. It is normal to feel a hole after the tooth is removed, and this will eventually heal completely, and bone will fill in the socket.

Diet

A nutritionally balanced diet is very important. During the first 24 hours, eat cool/room temperature soups and soft foods that are easily chewed and swallowed. You may gradually progress to solid foods over the next few days. Do not skip meals. By eating nutritious meals

regularly, you will feel better, gain strength, have less discomfort, and heal faster. Take any prescribed food supplement as directed. If you are a diabetic, maintain your normal diet and take medication as usual.

Nausea

Drink a carbonated beverage, such as ginger ale or cola, every 30 minutes until nausea subsides. You may also eat saltine crackers to help alleviate symptoms. If nausea persists, please call our office.

Dry Socket

When a dry socket occurs, there is constant pain that radiates to other areas of the jaw, teeth, and ear. Symptoms of a dry socket will not occur until the third or fourth post-operative day, and it results from a loss of the blood clot in the socket. This is similar to a scab pulling off the skin. All the surrounding areas will look normal. If you do not have improvement after the first few days following the procedure, please call the office. A medicated dressing may need to be placed to resolve the discomfort.

- as prescribed.
- While you are using narcotic pain medicine, do not drive, drink alcohol or perform any activity which requires attentiveness

Emergencies/Post-Op Info

Let Us Take Care of Your Dental Emergency

For patients who experience a dental emergency or trauma, we want to provide you with prompt, gentle emergency care Until you are in our office, though, you can refer to the general information below to best care for your child’s dental health

Chipped, Broken or Knocked-Out Tooth

The first 30 minutes after an accident are the most critical to treatment of dental trauma.

If your child’s primary tooth is knocked out:

- Never attempt to put a baby tooth back into its socket. Doing so may cause harm to the developing permanent tooth forming beneath the gum.

- Seek dental treatment as soon as possible to assess the socket and check for other any other injuries.
- Take the tooth with you to the pediatric dentist, if possible, so it can also be assessed.
- If you are in doubt whether it is a permanent or baby tooth, transport the tooth assuming it is permanent, covered in milk or saliva, **NOT** water.

If your child's permanent tooth is knocked out:

- Pick up the tooth by the crown part. Avoid touching the root of the tooth.
- If the tooth appears very dirty, wash it quickly with cold water for no more than 10 seconds.
- Holding the crown of the tooth, push the tooth back into the socket that it has come from. **If you feel comfortable doing so**
- If you do not want to put tooth back into socket: Seek dental treatment immediately. Timing is very important. A tooth that is replanted successfully within 60 minutes has a much greater chance of survival.
- Transport tooth covered in milk or saliva, **NOT** water.

What to do for a chipped or broken tooth:

- Seek dental treatment to fix the chip and check for any other oral injuries. If tooth fragment is found, place it in milk or saliva, **NOT** water.
- If the chip has exposed the pulp (nerve) of the tooth, it is at high risk of infection. The tooth needs to be patched up as soon as possible.
- If the trauma has made the tooth lose or moved it, the dentist may help to reposition and stabilize the tooth.

Emergencies Come With Delays

Care of Your Child's Mouth After Trauma

Please keep the area as clean as possible. A soft wash cloth works well during the healing process. Please follow these instructions:

- Watch for darkening of traumatized teeth
- Watch for infection (gum boils) in the area of trauma
- Maintain a soft diet for two to three days or until your child feels comfortable eating normally again
- Avoid sweets or foods that are extremely hot or cold
- If antibiotics or pain medicines are prescribed, be sure to follow the prescription as directed

Necessary Post-Operative Care

Care of the Mouth after Local Anesthetic

If the procedure was in the lower jaw, your child’s tongue, teeth, lip and the surrounding tissue will be numb or asleep. If the procedure was in the upper jaw, your child’s teeth, lip and the surrounding tissue will be numb or asleep.

Children do not understand the effects of local anesthesia, and may chew, scratch, suck or play with the numb lip, tongue or cheek. This can cause minor irritations or they can be severe enough to cause swelling and abrasions to the tissue. Watch your child closely for approximately two hours following the procedure. Keep your child on a liquid or soft diet until the anesthetic has worn off.

Extraction Post-Op Instructions

If your child has had one or more teeth “wiggled” out, the gauze needs to stay in place with biting pressure for 30 minutes, reducing the amount of bleeding.

Give your child the appropriate dose of children’s Tylenol, Motrin or Advil when you take the gauze out (no aspirin). Your child will need this for approximately 12 to 24 hours. If pain persists beyond 48 hours, call our office.

Your child should eat only soft, bland food for the first two days. Nothing sharp, crunchy or too hot or cold should be eaten because the area may be sensitive. Encourage plenty of liquids and let your child determine when a regular diet can be reintroduced. Spitting or drinking through a straw or “sippy” cup is not allowed. The force can start the bleeding again.

A clean mouth heals faster. Gentle brushing around the extraction site can be started immediately along with warm salt water rinses to aid with any discomfort.

Activity should be limited. Swelling after an extraction is common and should not cause alarm. If swelling occurs, apply an ice pack for 15 minutes on and 15 minutes off as needed in the 24 hours following tooth removal. Your child’s cheek, lip and tongue will be numb for approximately one to two hours. Be careful that your child does not bite at their cheek or pick at this area. As this area “wakes up,” it may feel funny. A self-inflicted bite injury is the most common post-op complication. Please monitor your child.

Care of Sealants

By forming a thin covering over the pits and fissures, sealants keep out plaque and food, decreasing the risk of decay. Since the covering is only over the biting surface

of the tooth, areas on the side and between teeth can't be coated with the sealant. Good oral hygiene and nutrition are very important in preventing decay next to these sealants or in areas unable to be covered.

Your child should refrain from eating ice or hard candy. This can fracture the sealant. Normal retention of a sealant is up to four years. Sealants that become displaced in the first 12 months will be replaced at no charge. After 12 months, sealants will be replaced at 50% charge for the next three years.

The American Dental Association recognizes that sealants can play an important role in the prevention of tooth decay. When properly applied and maintained, they can successfully protect the chewing surfaces of your child's teeth.

Regular visits to the dentist, the use of fluoride, daily brushing and flossing and limiting the number of times sugar-rich foods are eaten can help prevent tooth decay. If these measures are followed and sealants are used on your child's teeth, the risk of decay can be reduced or may even be eliminated.

Oral Discomfort After a Cleaning

A thorough cleaning can produce some bleeding and swelling and may cause some tenderness or discomfort. This is not due to a "rough cleaning," but due to tender and inflamed gums from insufficient oral hygiene. Alabaster Pediatric Dentistry recommends the following for two to three days after cleaning was performed:

- A warm salt water rinse two to three times per day (1 teaspoon of salt in 1 cup of warm water)
- For discomfort, use Children's Tylenol, Advil or Motrin as directed for the age of the child

Fluoride Varnish

- Your child should not brush or floss before bed so the fluoride can continue to soak into the teeth overnight
- Your child can eat and drink right away
  - Avoid crunchy (i.e. nachos) or sticky (i.e. gummies) foods for 24 hours, as these items can remove the fluoride from the tooth surfaces

↑ Back to top

Front Tooth Build-Up

- If your child required a tooth build-up due to a recent dental injury, please follow the post-op instructions for tooth trauma
- If your child was numbed for the procedure, please follow the post-op instructions for numbing
- The repair material is not as strong as natural tooth structure and prone to break off again
  - Avoid biting into hard foods with front teeth (such as nuts, carrots, hard candies)
- A mouthguard is recommended for contact sports
  - If you child would like a custom-fit mouthguard to improve comfort, speaking, and breathing, we can make one at our office
- If your child grinds his/her teeth while sleeping, a nightguard is recommended
- If the build-up falls off, place any pieces in a plastic bag and call the dentist to schedule an evaluation
  - If the build-up has fallen off several times, a dental crown may be indicated

↑ Back to top

Numbing (Local Anesthesia)

- The numbness will last from 1-4 hours
  - When your child states that the numb area is feeling "tingly" or "itchy," then the numbness is wearing off
- Avoid foods that requiring chewing
  - Ideally, wait until numbness wears off before feeding your child

    - If your child needs to eat, a soft food diet is recommended
- Monitor your child closely so he/she does not bite tongue, lips, cheeks, etc.
    - The numb sensation is typically the most challenging aspect for your child to understand
    - Sometimes, a distraction like a popsicle or rolled-up gauze can be helpful to prevent your child from traumatizing the numbed tissues

↑ Back to top

Pain Management

- If your child was numbed for his/her dental procedure, it is important to administer any pain medication before the numbing wears off
- For mild pain, have your child take acetaminophen (i.e. Tylenol) every 6 hours
- For moderate to severe pain, have your child alternate acetaminophen (i.e. Tylenol) and ibuprofen (i.e. Motrin) every 3 hours
    - Always follow the dosing instructions on the bottle(s) for your child's age and weight

↑ Back to top

Sealants

- Your child does not need to wait to eat or drink, as our sealant material is completely hardened or "set-up" at the end of the procedure

- Your child's bite may feel slightly different. This feeling will go away over the next 1-2 days
- Avoid chewing on hard candies or ice, as these foods can chip the sealants
  - If we notice any chipping or wear, we will discuss ways to repair the sealant

Temporary Sealants

- Occasionally, we will recommend temporary sealants if your child's tooth is at a higher risk for cavities and is not yet fully in the mouth
- This material is more saliva-resistant and takes time to fully harden.
- A soft food diet is recommended for 24 hours.
- When the tooth is fully in the mouth, we will replace the temporary sealant with a regular dental sealant

↑ Back to top

Separators

- Avoid activities that could cause the separator to be displaced
  - Avoid sticky foods (i.e. gummies, caramels, taffy, skittles, and gum) while the separators are in place
  - Avoid flossing in areas with separators
  - If a separator does become loose, please call our office so we can discuss options for replacement
- Expect your child's teeth to be mildly sore for the next 24 hours
  - Similar to braces, the separators cause the teeth to shift, which can cause soreness
  - Have your child take acetaminophen (i.e. Tylenol) or ibuprofen (i.e. Motrin) for discomfort

↑ Back to top

Silver Diamine Fluoride (SDF) Application

- Avoid eating or drinking for 30 minutes, this will allow the SDF to fully penetrate the treatment site
- The area with darken over the next 24 hours
  - If the soft tissues, such as the cheek or surrounding gums, accidentally contact the SDF, they will darken. This discoloration will go away completely within 10 days

↑ Back to top

Silver or White Crowns

- If your child was numbed for the crown procedure, please follow the post-op instructions for numbing
- Expect your child's gums to be sore for the next 2-3 days
  - When the crown is placed, it fits below the gumline, and the surrounding tissues need time to heal
  - You can help the area heal faster with gentle brushing and flossing
  - It is normal for the surrounding gums to appear discolored (i.e. red, purple, or white) or to bleed slightly during brushing
- Follow the post-op instructions for pain management to ensure your child is comfortable while healing
- It may take your child a few days to adjust to the crown. Do not let him/her pick at the area
- Avoid sticky or chewy foods (i.e. gummies, caramels, taffy, skittles, and gum) as they can cause the crown to become loose
  - If the crown falls out, save it in a plastic bag and call the office to have it re-cemented

↑ Back to top

Space Maintainers

- Expect your child's gums to be sore for the next 2-3 days
  - When a space maintainer is cemented, it fits below the gumline, and the surrounding tissues need time to heal
  - You can help the area heal faster with gentle brushing and flossing
- It may take your child a few days to adjust to the space maintainer. Do not let him/her pick at the area
- Avoid sticky or chewy foods (i.e. gummies, caramels, taffy, skittles, and gum) as they can cause the space maintainer to become loose
  - If the space maintainer falls out, save it in a plastic bag and call the office to have it re-cemented
  - If your child feels that the space maintainer is loose, please make sure the space maintainer is positioned correctly and call our office to schedule an assessment

↑ Back to top

Tooth-Colored Fillings

- If your child was numbed for the filling procedure, please follow the post-op instructions for numbing
- Our filling material is completely hardened or "set-up" at the end of the procedure, so your child only needs to wait to eat if he/she is numb
- For any discomfort, acetaminophen (i.e. Tylenol) or ibuprofen (i.e. Motrin) is recommended

- The tooth may be hypersensitive (have an increased sensation to hot, colds, or biting) for 1-2 weeks following the procedure
- Even though your child's cavity is gone, a new one could form
    - Fillings can get recurrent decay (a cavity that forms beneath the filling), and they should be brushed and flossed like a natural tooth

↑ Back to top

Tooth Removal (Extraction)

- Please follow the post-op instructions for numbing
- Your child should bite on a piece of gauze for 10-20 minutes following the procedure to stop the bleeding
    - Later in the day, when your child eats or brushes, it is not uncommon for the area to start bleeding again. If this happens, place another piece of folded gauze in the mouth and have your child bite and hold for 10-20 minutes
    - A small amount of oozing from the site for 1-2 days is normal. However, if the area continues to bleed excessively after biting on gauze for 10-20 minutes, call our office ASAP
- Avoid straws, spitting, sippy cups, bottles, and pacifiers for 24 hours
- Avoid hard, crunchy, or spicy foods for 24 hours
    - A soft food diet is recommended
- For any discomfort, acetaminophen (i.e. Tylenol) or ibuprofen (i.e. Motrin) is recommended
    - It is important for your child to start taking these medications before the numbing wears off in the next 1-4 hours
- For more information how to administer these medications for optimal pain management, please see our post-op instructions for "Pain Management"

↑ Back to top

Tooth Trauma

- Your actions may promote healing
  - Keep the area clean with gentle yet thorough oral hygiene, especially along the gumline
  - An antimicrobial mouth rinse may be prescribed to promote gum healing
  - A soft food diet is recommended for 10 days
  - Ibuprofen (i.e. Motrin) is recommended for 2 days for pain control and anti-inflammatory properties
- Any oral injuries could damage the nerve of the tooth
  - If the nerve does not heal, further treatment is required
  - Follow-up visits may be recommended so the dentist can make sure the area is healing appropriately
  - Please call our office if you observe any of the following: darkening of the tooth, increased pain or mobility, or abscess (gum pimple above the tooth)
- If the injured tooth is a baby tooth, there is a chance the developing permanent tooth also was injured
  - The best way to prevent further injury is to follow-up with the dentist as recommended
- If age-appropriate, have your child wear a mouthguard for contact sports
  - If you child would like a custom-fit mouthguard to improve comfort, speaking, and breathing, we can make one at our office

↑ Back to top

Dental Surgery Under General Anesthesia

- If your child was asleep for his/her dental treatment, there are several steps to help your child’s recovery

- Sore throat and irritability are common following dental surgery
  - Follow the post-op instructions for pain management
- Nausea is common immediately following dental surgery
  - First, give your child clear liquids (water, apple juice, clear broth). If your child can keep these down, you may move to bland foods (yogurt, crackers, rice)
  - A soft food diet is recommended for the first 24 hours
- Give your child plenty of fluids over the next 24 hours
  - A slightly elevated temperature after surgery is often due to dehydration and be controlled by increasing fluid intake or by taking ibuprofen (i.e. Motrin) or acetaminophen (i.e. Tylenol)
  - If a significant fever (>101.4oF) occurs, call our office immediately or go to your local emergency room
- Do not brush or floss the teeth until tomorrow as the mouth will be sore tonight
  - This will also allow the fluoride varnish treatment soak into the teeth overnight
  - You may resume your normal brushing and flossing routine tomorrow morning
- If your child is having issues such as a fever (>101.4oF), bleeding, or other medical problems related to dental surgery, call our office immediately go to your local emergency room
- Please return for your follow-up appointment so we can ensure your child's mouth is and healing normally

Emergencies & Post-Op Instructions

Emergencies
If your child has an accident, please call our office as soon as possible. If it is an after-hours emergency, a pager number will be given on the answering machine.

The first 30 minutes after an accident are the most critical to treatment of dental trauma. If a permanent tooth is knocked out, gently rinse, but do not scrub the tooth under water. Replace the tooth in the socket if possible. If this is impossible, place the tooth in a glass of milk or a clean wet cloth and come to the office immediately. If the tooth is fractured, please bring in any pieces you can find.

Our schedule may be delayed in order to accommodate an injured child. Please accept our apologies in advance should an emergency occur during your child's appointment. We will provide you the same care should your child ever need emergency treatment.

Care of the Mouth after Trauma
Please keep the traumatized area as-clean-as possible. A soft wash cloth often works well during healing to aid the process.

Watch for darkening of traumatized teeth. This is a common result following trauma and could be an indication of a dying nerve (pulp). If the swelling should re-occur, our office needs to see the patient as-soon-as possible. Ice should be administered during the first 24 hours to keep the swelling to a minimum.

Watch for infection (gum boils) in the area of trauma. If infection is noticed – call the office so the patient can be seen as-soon-as possible.

Maintain a soft diet for two to three days, or until the child feels comfortable eating normally again.

Avoid sweets or foods that are extremely hot or cold.

If antibiotics or pain medicines are prescribed, be sure to follow the prescription as directed.

Post-op Instructions

- Extractions
- Care of Sealants
- Oral Discomfort After Cleaning
- Post-Sedation Instructions

Care of the Mouth after Local Anesthetic
Your child has had local anesthetic for their
dental procedure:

- If the procedure was in the lower jaw… the tongue, teeth, lip and surrounding tissue will be numb or asleep.
- If the procedure was in the upper jaw… the teeth, lip and surrounding tissue will be numb or asleep.

Often, children do not understand the effects of local anesthesia, and may chew, scratch, suck, or play with the numb lip, tongue, or cheek. These actions can cause minor irritations or they can be severe enough to cause swelling and abrasions to the tissue. Please monitor your child closely for approximately two hours following the appointment. It is often wise to keep your child on a liquid or soft diet until the anesthetic has worn off.

Extraction Post-op Instructions
1. Your child has had 1 or more teeth "wiggled" out.

2. The gauze needs to stay in place with biting pressure for 30 minutes. This will reduce the amount of bleeding.

3. Give your child the appropriate dose of children's Tylenol, Motrin or Advil when you take the gauze out (NO aspirin). Your child should only need this for approximately 12 to 24 hours. If pain persists beyond 48 hours, please call our office.

4. Your child should eat only soft, bland food for the first couple days- nothing sharp, crunchy or too hot or cold because the area may be a sensitive. Encourage plenty of liquids (water, soups, juices, etc.). Let your child determine when a regular diet can be reintroduced.

5. NO spitting or drinking through a straw or “sippy” cup. The force can start the bleeding again.

6. A clean mouth heals faster. Gentle brushing around the extraction site can be started immediately along with warm salt water rinses (1/4 teaspoon to a glass of water) to aid with any discomfort.

7. Activity may need to be limited. Sometimes a nap is a good idea.

8. Swelling after an extraction is not uncommon and should not cause alarm. If this occurs, apply an ice pack for 15 minutes on and 15 minutes off as needed in the 24 hours following tooth removal.

9. Your child’s cheek, lip and tongue will be numb for approximately 1-2 hours. Please be very careful that your child does not bite at his/her cheek or pick at this area. As this area “wakes up” it may feel funny. A self-inflicted bite injury is the most common post-op complication. Please keep on eye on your child!

Care of Sealants
By forming a thin covering over the pits and fissures, sealants keep out plaque and food, thus decreasing the risk of decay. Since, the covering is only over the biting surface of the tooth, areas on the side and between teeth cannot be coated with the sealant. Good oral hygiene and nutrition are still very important in preventing decay next to these sealants or in areas unable to be covered.

Your child should refrain from eating ice or hard candy. This tends to fracture the sealant. Normal retention of a sealant is up to four years. Sealants that become displaced in the first twelve months will be replaced at no charge. After twelve months, sealants will be replaced at 50% charge for the next 3 years.

The American Dental Association recognizes that sealants can play an important role in the prevention of tooth decay. When properly applied and maintained, they can successfully protect the chewing surfaces of your child’s teeth. A total prevention program includes regular visits to the dentist, the use of fluoride, daily brushing and flossing, and limiting the number of times sugar-rich foods are eaten. If these measures are followed and sealants are used on the child’s teeth, the risk of decay can be reduced or may even be eliminated!

Oral Discomfort after a Cleaning
A thorough cleaning unavoidably produces some bleeding and swelling and may cause some tenderness or discomfort. This is not due to a “rough cleaning”, but to tender and inflamed gums from insufficient oral hygiene. We recommend the following for 2-3 days after cleaning was performed:

1) A warm salt water rinse 2 – 3 times per day
(1 teaspoon of salt in 1 cup of warm water)

2) For discomfort use Children’s Tylenol, Advil, or Motrin as
directed for the age of the child.

Post-Sedation Instructions
It is important for your child’s safety that you follow these instructions carefully! Failure to follow these instructions could result in unnecessary complications.

- **ACTIVITIES:** Do NOT plan or permit activities for your child after treatment. Allow your child to rest. Closely supervise any activity for the remainder of the day. When sleeping, encourage your child to lie on his/her side or stomach.

- **GETTING HOME:** Two responsible adults must accompany your child. One adult should drive your child home and a second responsible adult must be available to take care of your child while driving home. Your child should be closely watched for signs of breathing difficulty and carefully secured in a car seat or seat belt during transportation.
- **DRINKING or EATING after TREATMENT:** After treatment, the first drink should be plain water. Clear liquids can be given next (fruit juice, sugar-free Kool-Aid, Gatorade, ginger ale, soup broth, etc). Small drinks taken repeatedly are preferable to taking large amounts. Soft, luke-warm, bland food may be taken when desired (mashed potatoes, yogurt, soup, pudding, ice cream, popsicles, etc.).
- **TEMPERATURE ELEVATION:** Your child's temperature may be elevated to 101 F (38 C) for the first 24 hours after treatment. Tylenol every 3-4 hours and fluids will help alleviate this condition. Temperature above 101° F (38° C) is cause to notify Dr. Dave and your pediatrician.
- **EXTRACTIONS:** If your child had teeth removed, a small amount of bleeding is normal. Do NOT let your child spit, as this will cause more bleeding. In order to not disturb the blood clot, do NOT use a straw to drink for the first 24 hours. Also, remember that a small amount of blood mixed in with a lot of spit in the mouth looks like a lot of blood.
- **BRUSHING:** Since your child has had a cleaning and fluoride treatment today, it is not necessary to resume tooth brushing and flossing until tomorrow morning. However, it is very important for you to brush and floss your child's teeth beginning tomorrow morning and on a daily basis thereafter, to prevent infection and future dental problems.
- **SEEK ADVICE:** If any of the following problems arise, call Raleigh Pediatric Dentistry, or if the office cannot be reached, call the Emergency Department at your local hospital:

· If vomiting persists beyond four (4) hours

· If the temperature remains elevated beyond 24 hours or goes above 101° F (38° C).

· If there is any difficulty breathing or coloration of the skin is poor.

Post-Op Instructions Following Root Canal Therapy

Please follow the guidelines below and contact us with any questions.

- No eating or drinking for the first 30 minutes following your treatment. Also avoid feeling around your tooth with your tongue. This is to allow the temporary filling sufficient time to harden.
- If you were given any prescriptions, please have them filled promptly and take as directed. If no prescriptions were given, you may choose to take ibuprofen medications such as Motrin or Advil (as long as you do not have any allergies to these medications) for alleviation of discomfort and swelling. Alternatively, you may use Tylenol. Do not exceed the guidelines printed on the label for any medication. If you are unable to achieve adequate pain control, please call our office.
- Applying a cold compress to the face near the treatment area will help minimize swelling. If using an ice pack, please do not apply ice directly to your skin but place a cloth between the

ice and your skin at all times. You may apply the cold compress for up to 20 minutes on and at least 20 minutes off for the next 6-8 hours as needed.

- Once you resume eating and drinking, avoid chewing or biting on the treatment area until your permanent restoration is placed. The temporary filling or crown placed immediately following root canal treatment is usually a soft composite that is vulnerable to fracturing (cracking). For this reason, it is important to avoid chewing on hard substances such as peanuts, pretzels, hard candy, ice cubes, etc.. You may experience increased sensitivity prior to the placement of the final restoration. You will need to see a restorative dentist within a month to have a permanent crown or filling placed. Please contact your restorative dentist to make an appointment at your earliest convenience. Waiting longer than a month increases the chances that the temporary will fracture or that decay will develop in the affected area.
- Please keep the treated area clean by gently brushing and flossing regularly.
- It is rare for a temporary filling to fall out although it may divot while in use. If the temporary falls out, please contact your general dentist as soon as possible. If your temporary falls out after office hours, you may purchase some temporary filling material from a pharmacy and follow the included instructions to cover the area until you can be seen in our office.
- Some discomfort is normal for 2 to 4 days following root canal therapy. In some cases the tooth and surrounding tissue may be sore for a few weeks following treatment.
- Please brush and floss as usual unless otherwise instructed by our office. Follow any other instructions provided by our office during your visit. Please take all medications as prescribed.

THIS SECTION IS VERY IMPORTANT, PLEASE READ CAREFULLY

While flare-ups are rare, they occur in about 5% of cases and may cause significant pain. They generally only occur with teeth that are extremely irritated and/or infected or with teeth that have a history of prior treatment. These sometimes occur randomly, even on patients that have had root canals done in the past without problems. If you have a flare-up, you may experience moderate to severe pain, swelling, throbbing, or general discomfort; please contact our office right away. You may be prescribed additional medication such as antibiotics, and/or you may be asked to come to the office for further treatment.

Post Op Instructions: After a Root Canal Root canal therapy can take 1-2 appointments to complete. After each appointment when anesthetic has been used, your lips, teeth and tongue may be numb for several hours. Avoid any chewing on the side of the treated tooth until the numbness has completely worn off. A temporary filling or crown is placed by your dentist to protect the tooth between appointments. Between appointments, it's common (and not a problem) for a small portion of your temporary filling or crown to wear away or break off. If the entire filling falls out, or if a temporary crown comes off, please call our office so it can be replaced. To protect the tooth and help keep your temporary in place: ·Avoid chewing sticky foods

(especially gum). ·Avoid biting hard foods and hard substances, such as ice, fingernails and pencils. ·If possible, chew only on the opposite side of your mouth. It's normal to experience some discomfort for several days after a root canal therapy appointment, especially when chewing. It is not uncommon for a tooth to be uncomfortable or even exhibit a dull ache immediately after receiving root canal therapy. This should subside within a few days (or even weeks). Even if you were not experiencing any discomfort prior to treatment, it is normal for you to experience some degree of discomfort for a few days after. The tenderness is normal and is no cause for alarm. To control discomfort, take pain medication as recommended by your dentist. If antibiotics are prescribed, continue to take them for the indicated length of time, even if all symptoms and signs of infection are gone. If you were not prescribed a pain medication by us but are experiencing pain after your appointment, we recommend taking over the counter pain medication. We recommend ibuprofen (Motrin, Advil, Nuprin) or naproxen (Aleve, Anaprox). Should you experience discomfort that cannot be controlled with pain medications or should swelling develop, please call our office. To further reduce pain and swelling, rinse three times a day with warm salt water; dissolve a teaspoon of salt in a cup of warm water, then rinse, swish, and spit. It's important to continue to brush and floss normally. Usually, the last step after root canal treatment is the placement of a crown on the tooth. A crown covers and protects the tooth from breaking in the future. Unless otherwise noted by the dentist, it is critical to have a crown placed on your root canal therapy treated tooth as soon as possible. Delay in obtaining final restoration (crown) may result in fracture and/or possible loss of the tooth. If your bite feels uneven, you have persistent pain, or you have any other questions or concerns, please call our dental office.

Post Treatment Care

After your procedure, your endodontist will send you home with instructions for pain management and how to care for your tooth while recovering from treatment and until

a follow-up visit. Following guidelines for care is especially important if a temporary filling or crown is in place.

As the medication used to numb your mouth during the procedure wears off, you may feel some tenderness in the area for a few days as everything heals and some mild soreness in your jaw from keeping your mouth open for an extended period during the procedure. These temporary symptoms usually respond well to over-the-counter medication but your doctor may prescribe stronger, narcotic medication as well. It’s important to carefully follow the instructions for medications and that narcotics can make you drowsy so you should exercise caution when taking them and driving a car or operating dangerous machinery.

Though you may experience a slightly different sensation from your treated tooth than your other teeth for some time, you should contact your endodontist immediately if you experience any of the following symptoms:

- Severe pain or pressure lasting more than a few days
- Visible swelling inside or outside your mouth
- An allergic reaction to medication (rash, hives or itching)
- Your bite feels uneven
- The temporary crown or filling, if one was put in place, comes out (losing a thin layer is normal)
- Symptoms you experienced prior to treatment return

Root Canals Explained

After a root canal, it's important to take special care of the affected tooth for a few weeks or until your tooth is fully restored by your endodontist or dentist recommends otherwise. You can brush and floss as usual, but make sure to be gentle around the treated area. Avoid chewing on hard foods or using the treated tooth for biting down heavily until you've been cleared by your endodontist or dentist.

You should not chew, drink hot or cold liquids, or smoke for the first hour. Additionally, smoking can interfere with the healing process and should be avoided.

Once your root canal and any follow-up appointments are completed, **you’ll need to return to your dentist for a final crown to fully restore the tooth**. It’s important to make this appointment as soon as your endodontist completes work on your tooth. A properly treated and restored tooth can last as long as your natural teeth.

Take care of your teeth by brushing, flossing, regular checkups and cleanings and be sure to return to your dentist and/or endodontist if you experience pain or swelling in the future. It's possible for a properly treated tooth to require treatment again even years after a first procedure but often when this occurs the tooth can be saved.

Pain Management

Root canal treatment means the end of pain, not the beginning of it. Anyone who's ever suffered from pulp inflammation or infection knows the hurt of toothache, which only gets worse the longer it goes untreated. Even taking a sip of water can be a painful experience to someone in need of root canal treatment.

An endodontist is well aware of the painful plight of toothache, and they are experts in pain management. An endodontist makes patient comfort a priority and strives to keep procedures pain free, with advanced numbing techniques and a gentle bedside manner.

With modern techniques and anesthetics, most patients report that they are comfortable during the procedure.

For the first few days after treatment, your tooth may feel sensitive, especially if there was an infection before the procedure. This discomfort can be relieved with over-the-counter or prescription medications. Follow your endodontist's instructions carefully.

When is it time to see an endodontist?

No one enjoys having work done on their teeth and some even delay their bi-annual cleanings out of fear or reluctance to visit the dentist. But when it comes to real tooth pain, it's important to take care of it as soon as possible. You'll feel better and likely save your tooth.

If you're experiencing tooth pain, have injured your tooth, feel sensitivity to hot or cold, and/or there is swelling around the teeth, gums or your face, make an appointment with an endodontist or pay a visit to your dentist, first.

Your dentist may refer you to an endodontist — an expert at saving teeth with two or more years of specialized training above and beyond dental school.

Always keep in mind that beginning your root canal procedure means ending your tooth pain.

Periapical surgery, periradicular surgery post op instructions

Please read the following instructions carefully   On the day of your operation Until the local anaesthetic has worn off, please take care when eating/drinking.   Keep a soft light diet.   Avoid hot food/drink.   Avoid alcohol.   Avoid smoking.   Avoid excessive exertion.   Do not rinse/spit.   Avoid touching/pulling soft tissues around the surgical site.   Avoid chewing directly on the treated tooth/ teeth for 5 days.   There may be a little oozing/bleeding following surgery. If this is the case then apply gentle pressure to the wound site with a clean rolled cotton handkerchief/gauze for 15 minutes. If oozing/bleeding continues after this then please contact us immediately.   Pain/Swelling Relief   Pain relieving tablets such as Paracetamol or Ibuprofen can be taken in conjunction with each other and following manufacturer’s instructions. This can be commenced before the local anaesthetic wears off.   Avoid using hot packs on the outside of your mouth.   Ice packs can be used if swelling occurs. Use them for 5 minutes for up to 5 times for the first 24 hours.   TIG 18/17 Updated: Jan 2019 Review: Date Jan 2022   Page 2 of 2   www.mft.nhs.uk   Aftercare   The day after your surgery start using a mouthwash of salt water following each meal. (Add 1 teaspoon of salt to 1 cup of boiled water. Allow to cool before use)   Do not brush the treated area for 7 days.   During this period of 7 days it is advised to use an anti-bacterial/Chlorhexidine mouthwash three times per day.   Sutures/stitches   Reabsorbable (dissolvable) sutures are mostly used for this type of procedure.   Non-reabsorbable sutures may be used in specific cases and will be removed in the surgery at your review appointment.   Follow up appointments are routinely made in order to monitor and record surgery recovery progress. If you have any concerns/ queries about the information contained within this leaflet or any other aspects of your treatment, please do not hesitate to contact us:

INSTRUCTIONS FOR AFTER ENDODONTIC SURGERY Ice: The day of surgery, after the procedure, continue icing the area (15 minutes on, 15 minutes off) for the next two hours. Rest: After your surgical treatment, rest as much as possible for the next 24hrs. Do not attempt physical exertion at work or play, for these activities will raise blood pressure resulting in increased bleeding, swelling and pain. Avoid bending over and lifting heavy items the first day. Your discomfort is generally minimal after the first 12 hours. However, there is a wide range of healing patterns among individuals. While sleeping or resting, place pillows behind your head and neck to slightly elevate your head. You may want to protect your pillow using an old towel or pillow case. Do Not: Raise your lip to look at the sutures. This may disrupt healing or tear the sutures. Avoid alcohol for the first 24 hours Eating: Eat what is comfortable for you. Try to avoid eating brittle, crunchy food as well as hard foods that require heavy pressure to chew. Soft foods and liquids are best and avoid chewing on the side that was treated. Smoking: Please refrain from smoking for the first 48 hours after surgery. Sucking or "dragging" on a cigarette causes a negative pressure in the mouth and enhances bleeding. The chemicals in smoke can delay healing. Mouth Rinse: AVOID any vigorous rinsing or spitting for the first 24hrs to ensure proper healing and clot formation. Start the night of surgery, and continue using the mouth rinse for the next two weeks. (A.M. and P.M.) Brushing: Maintain your normal oral hygiene program of brushing and flossing EXCEPT in the surgical area. Do not floss the surgical area for 10 days. Avoid brushing the surgical site for 1 week. After 3-4 days you can gently lift the gum and clean the teeth in the surgical site using a q-tip dipped in the mouth rinse. Discoloration and Bleeding: It is normal to have some discoloration of the tissues around the surgical site. You may notice some slight bleeding at the surgery site after treatment. Discoloration (bruising) of the skin or cheek under the jaw line may occur occasionally. This does not occur with every patient, but if it does, there is no concern. Pain and Swelling: Do not be alarmed if you develop swelling. Swelling is a normal consequence of manipulating the tissues at the surgical site. It is important to understand that there is a wide range of healing patterns following a surgery. Some individuals have very little pain or swelling the next day. Other people can have more intense pain and swelling for several weeks. Regardless of where you fall on this scale, you can expect to still have some tenderness in that area a week or more after the surgery because it takes time for tissues to

heal. It typically takes several weeks for gums to feel normal. It may take the tooth several months to feel completely normal as your body fills in the bone around the tooth. Sutures: The sutures we placed may begin to fall out or dissolve prior to your post op appointment, this is normal.

How Do I Stay Healthy After Endodontic Treatment?

After Initial Treatment

The tooth and its surrounding gum tissues may be somewhat tender for several days as a result of manipulation during treatment and also because of the condition with which you came to the office.

If necessary, take pain medication every four to six hours and bathe the tooth with warm salt water. A level teaspoonful of ordinary salt placed in a glassful of tap water, as hot as you can comfortably tolerate, is the right combination. For faster relief, bathe the tooth every hour on the hour.

Do not be surprised if you cannot eat with this tooth for a few days. Brush your teeth as usual

After Endo Treatment

Endodontic treatment for this tooth has now been completed. The root canal, or canals, have been permanently sealed. However, the outer surface is sealed only with a temporary cement. PLEASE PHONE YOUR DENTIST FOR AN APPOINTMENT- A PERMANENT RESTORATION MUST BE PLACED TO PROTECT YOUR TOOTH AGAINST FRACTURE AND DECAY.

Please phone our office for a re-examination appointment in one year. X-rays taken then will permit us to review the healing process following your treatment. THERE IS NO CHARGE FOR THIS SERVICE. It is important that endodontically treated teeth be re-examined regularly.

Your dentist will be informed that your endodontic treatment has now been completed and a progress report will be sent to him/her after each examination.

After endodontic periapical or radicular Surgery

WHAT TO DO FOLLOWING SURGERY:

1. After leaving this office, rest and avoid strenuous activities for the remainder of the day.
2. If necessary, take pain medication for relief of discomfort.
3. Apply an ice bag or cold compress to the outside of your face over the operated area. Apply for 10 minutes, then remove for 5 minutes. Continue the applications for the first 4–6 hours of the first day only. This will help to keep swelling and discolouration to a minimum.
4. Eat soft foods only for the first 24 hours.
5. Avoid chewing in the operated area for the first 24 hours.
6. Do not brush the operated area for 1-2 days.
7. After 24 hours, take gentle mouth rinses using plain warm water following meals.
8. Avoid lifting or unnecessary exercising of your lip. It is possible to accidentally tear the sutures, open the incision and delay healing.

WHAT TO EXPECT FOLLOWING SURGERY:

1. Some discomfort will usually be present. Severe pain seldom occurs. If Aspirin, Tylenol, ibuprophen, etc., do not restore comfort, please telephone for prescribed pain reliever.
2. There will be some swelling and discolouration present for 3-5 days following surgery. This is a normal part of the healing process. Swelling reaches a maximum on the second morning following the surgery.
3. There is often a temporary loss of feeling in the operated area.
4. The operated tooth may feel loose for a time.
