# Supplementary material for: Leveraging Large Language Models in the delivery of post-operative dental care: a comparison between an embedded GPT model and ChatGPT
Source: BDJ Open. 2024 Jun 12;10:48. doi: 10.1038/s41405-024-00226-3 (PMC11169374; doi:10.1038/s41405-024-00226-3)
Supplement: Supplementary file 2 — Supplementary Note 2 [file 41405_2024_226_MOESM2_ESM.pdf]

Questions and responses generated via both models for four different specialties of dentistry

*The responses generated via embedded GPT model and chatGPT were assigned codes "A" or "B", respectively.

Periodontics

1. Can I eat or drink immediately after scaling?

Code A: You may start with non-abrasive foods such as mashed potatoes, pasta, cottage cheese, soup, or scrambled eggs as soon as the local anesthetic wears off. However, it is advised to avoid sticky things for the first 24 hours. After that, you may eat, drink, and clean your tooth just as you do regularly.

Code B: You should wait at least 30 minutes after scaling to eat or drink. This allows your gums and teeth to recover from the procedure. Drinking water is fine, but avoid hot, cold, or acidic beverages.

2. My teeth were loose after scaling; is it normal?

Code A: It is normal to experience some tooth sensitivity and mild looseness after scaling. This should subside within a few days as the gums heal. If the looseness persists or worsens, it is recommended to contact your dentist for a follow-up evaluation

Code B: Slight mobility or looseness in your teeth after scaling is normal. It should improve within a few days as your gums heal. If it persists or worsens, consult your dentist for a follow-up.

3. When can I resume my regular oral care routine, including brushing and flossing?

Code A: You can resume your regular oral care routine, including brushing and flossing, after scaling. It is important to continue practicing good oral hygiene to maintain the health of your teeth and gums. However, be gentle while brushing and flossing around the treated areas to avoid any discomfort or irritation.

Code B: You can resume your regular oral care routine, including brushing and flossing, the day after scaling. However, be gentle around the treated areas to avoid any discomfort. If your dentist provided specific instructions, follow them.

4. Is it normal to experience bleeding or swelling after the procedure?

Code A: It is normal to experience some minor bleeding and swelling after the procedure. This should subside within a few days as the gums heal. To manage bleeding, apply gentle pressure with a clean gauze pad or a moistened tea bag. To reduce swelling, you can apply ice packs to the outside of your face for 20 minutes on and 20 minutes off during the first 48 hours. If bleeding or swelling persists or worsens, it is advisable to contact your dentist for further evaluation.

Code B: It's normal to experience minor bleeding and swelling after scaling. This should subside within a day or two. If bleeding and swelling persist or worsen, consult your dentist.

5. My gums are going down and my lower teeth are getting more sensitive; can I do something to correct it?

Code A: If you are experiencing receding gums and increased tooth sensitivity, it is important to consult with your dentist. They can evaluate the condition of your gums and teeth and recommend appropriate treatment options, such as desensitizing toothpaste, fluoride treatments, or gum grafting, to address the issue. It is best to seek professional advice for an accurate diagnosis and personalized treatment plan.

Code B: Receding gums and increased tooth sensitivity may be due to gum disease. It's important to see a dentist for an evaluation and possible treatment. Maintaining good oral hygiene and using a soft toothbrush can help prevent further issues.

6. Can I smoke or use tobacco products after scaling?

Code A: It is strongly advised to avoid smoking or using tobacco products for at least 72 hours following scaling. Smoking can delay the healing process and increase the risk of complications. It is best to refrain from smoking to promote optimal healing and oral health.

Code B: It's best to avoid smoking or using tobacco products after scaling. Tobacco can slow down the healing process and increase the risk of complications. It's a good opportunity to consider quitting for better oral health.

7. What over-the-counter pain relievers or medications can I take if needed?

Code A: You can take over-the-counter pain relievers such as ibuprofen (Advil, Motrin) or acetaminophen (Tylenol) as directed on the bottle to relieve any discomfort after scaling. However, it is always recommended to consult with your dentist or pharmacist before taking any medication to ensure it is safe for you and does not interact with any other medications you may be taking.

Code B: You can take over-the-counter pain relievers like ibuprofen or acetaminophen, following the recommended dosage, if you experience any discomfort after scaling. Consult with your dentist or a healthcare professional for specific advice based on your situation.

8. Which toothpaste should we use after scaling?

Code A: After scaling, it is recommended to use a toothpaste that is specifically formulated for sensitive teeth. These toothpastes contain ingredients that help to reduce tooth sensitivity and provide relief. Your dentist or dental hygienist can recommend a suitable toothpaste for you based on your specific needs.

Code B: Use a toothpaste that is specifically designed for sensitive teeth. Look for one with fluoride to help prevent cavities. Your dentist may also recommend a suitable toothpaste based on your individual needs.

9. Should we use mouth rinses after scaling and for how many days?

Code A: After scaling, you can use a mouth rinse to aid in the healing process. It is recommended to use a non-alcohol-based mouthwash or a saline solution (warm saltwater rinse) for the first week after scaling. Rinse gently with the mouthwash or saline solution 3-4 times a day but avoid swishing or gargling vigorously. It is best to follow the instructions provided by your dentist or dental hygienist for the specific duration of mouth rinse use.

Code B: You can use a non-alcoholic, antimicrobial mouth rinse after scaling, but it's best to wait for 24 hours to allow your gums to heal. Follow your dentist's recommendations on the duration of use, which may vary depending on your specific case.

10. The tongue feels weird when it touches the lower anterior teeth after scaling

Code A: It is normal to feel a slight difference in texture or contour of the teeth after scaling. Your tongue may magnify this small difference, causing a strange sensation when it touches the lower anterior teeth. However, you should become accustomed to this sensation within a few days. If the feeling persists or worsens, it is advisable to contact your dentist for a follow-up evaluation.

Code B: It's common to feel a difference in texture on your teeth and tongue after scaling. This sensation should improve as your mouth adjusts. If it persists or becomes uncomfortable, consult your dentist for advice.

Oral and Maxillofacial Surgery

1. When can I resume normal daily activities after the extraction of teeth?

Code A: After the extraction of teeth, it is recommended to limit your activities on the day of the surgery. You can resume your normal daily activities the following day, but it is important to avoid any strenuous activities or heavy lifting for at least 48 hours to promote proper healing.

Code B: You can generally resume normal daily activities the day after a tooth extraction. However, it's important to follow your dentist's post-extraction care instructions to ensure proper healing and avoid complications.

2. How long does it bleed after dental extraction?

Code A: Minor bleeding is expected after dental extraction surgery and it will usually subside quickly and stop within an hour or two after the surgery. However, some oozing may persist for several hours. If bleeding persists after 4 hours, it is recommended to wet a tea bag, wrap it in gauze, and bite on it without changing for 2 hours.

Code B: Bleeding after a dental extraction can vary, but it typically lasts for a few hours to a day. If it continues beyond 24 hours or is excessive, contact your dentist for guidance.

3. When do I visit the clinic to remove the sutures after surgical extraction?

Code A: The sutures used during surgical extraction are typically dissolvable and will fall out on their own within 7-10 days. If the sutures need to be removed, you will be notified by the clinic and an appointment will be made for you. It is important to follow up with the clinic for periodic check-ups until the implant has fully healed.

Code B: Sutures are typically removed 5-7 days after a surgical dental extraction. Your dentist will provide specific instructions based on your case.

4. How long do I have to avoid smoking after extraction?

Code A: It is strongly advised to avoid smoking for at least 72 hours (3 days) after dental extraction surgery. Smoking can delay the healing process and increase the risk of

complications such as dry socket. It is best to refrain from smoking to promote optimal healing.

Code B: To promote healing, it's best to avoid smoking for at least 48 hours after a dental extraction. However, quitting smoking altogether is the healthiest choice for your oral and overall well-being.

5. Is it safe to brush and floss my teeth immediately at the extraction site?

Code A: No, it is not safe to brush or floss the extraction site immediately after the surgery. It is important to wait for 24 hours before brushing your teeth. After 24 hours, you can gently brush your teeth using a soft toothbrush, but avoid the extraction site. It is also recommended to rinse your mouth with warm salt water after each meal to help soothe discomfort.

Code B: After a dental extraction, it's best to avoid brushing and flossing the extraction site for the first 24 hours. This allows the blood clot to form and the area to heal. Follow your dentist's instructions for when to resume regular oral care.

6. I have noticed bruising on my face after I got my extraction done yesterday, is it normal?

Code A: Yes, it is normal to experience some bruising on the face after dental extraction surgery. Bruising is a common response and should resolve within 7-14 days after the surgery. To help minimize bruising, you can apply ice packs to the outside of your face in intervals of 20 minutes on and off for the first 24 hours following surgery.

Code B: Some bruising and swelling after a dental extraction can be normal, especially for surgical extractions. This should improve in a few days. Use a cold compress and follow any post-operative care instructions provided by your dentist.

7. How long does it take for the minor oozing to stop after a dental extraction?

Code A: Minor oozing is normal for up to 24 hours after a dental extraction. However, if you are taking blood thinners, the oozing may last longer. If the oozing continues beyond 24 hours or if it becomes excessive, it is recommended to follow the instructions provided and contact your dental office for further guidance.

Code B: Minor oozing or bleeding after a dental extraction typically stops within a few hours. If it continues or worsens, gently biting on a clean gauze pad can help control it. If it persists beyond 24 hours, contact your dentist.

8. Can I have tea or soup right after extraction?

Code A: After a dental extraction, it is recommended to stick to a strictly liquid diet until your jaw is no longer tightly held together. During this period, you can consume cool/room temperature soups and soft foods that are easily chewed and swallowed. It is important to avoid hot foods and drinks, including tea, immediately after the extraction to prevent any complications.

Code B: After a dental extraction, it's best to avoid hot beverages and soup for the first 24 hours, as they can disrupt the healing process. Stick to lukewarm or cold liquids and soft foods during this time.

9. Is it mandatory to take antibiotics after each extraction?

Code A: Antibiotics are not always necessary after each extraction. They are typically prescribed in specific cases, such as when there is a risk of infection or if you have a weakened immune system. It is important to follow your dentist's instructions and take antibiotics as prescribed if they are recommended for your specific situation.

Code B: Antibiotics are not mandatory after every dental extraction. Your dentist will prescribe them only if there's a risk of infection or if you have a pre-existing condition that requires them. Always follow your dentist's recommendations regarding antibiotics.

10. What is the sequence of warm and cold compresses after extraction?

Code A: After a dental extraction, it is recommended to apply cold compresses to the surgical area (side of the face) for the first 48 hours. You can apply ice packs for 20 minutes on and 10 minutes off. After 72 hours, you can switch to warm compresses to help relieve swelling. Remember to always use a cloth between the compress and your skin to prevent direct contact.

Code B: Apply a cold compress for the first 24 hours to reduce swelling, then switch to a warm compress after that to ease discomfort. Use each compress for about 15-20 minutes at a time, with breaks in between.

Restorative Dentistry & Endodontics

1. How long would it take for the anesthesia to subside after restorative work?

Code A: The duration for the anesthesia to subside after restorative work can vary, but it typically lasts for a few hours. It is important to be cautious during this time to avoid accidentally biting your lips, cheeks, or tongue. Once the anesthesia wears off, you can resume your normal eating habits.

Code B: Anesthesia effects typically subside within 2-4 hours after restorative dental work. Avoid chewing or eating until the numbness is completely gone to prevent accidentally biting your cheek or tongue.

2. Is it safe to brush or floss teeth right after a filling or root canal?

Code A: Yes, it is safe to brush and floss your teeth after a filling or root canal. However, it is important to wait until the anesthesia has completely worn off to avoid accidentally injuring your gums or soft tissues. Gentle brushing and flossing can help maintain oral hygiene and promote healing after the procedure.

Code B: You can usually resume brushing and flossing your teeth after a filling or root canal. However, be gentle around the treated area. If your dentist provides specific instructions, follow them carefully.

3. How long should I wait before eating from the side where the silver filling (amalgam) was done?

Code A: For silver fillings (amalgam), it is generally recommended to wait for 2 to 3 hours before eating on the side where the filling was done. Amalgam fillings set up hard right away, so you can resume normal eating habits after this waiting period. However, it is always best to follow the specific instructions provided by your dentist.

Code B: You can eat on the side where an amalgam filling was placed as soon as the anesthesia wears off. Unlike some other dental materials, amalgam fillings harden quickly and are safe for immediate use.

4. How long should I wait before eating from the side where the tooth-colored filling (composite) was done?

Code A: You can eat on the side where the tooth-colored filling (composite) was done as soon as the anesthetic wears off. Just be careful not to bite your lips, cheeks, or tongue until the anesthetic completely wears off.

Code B: You can eat on the side where a tooth-colored composite filling was placed immediately after the procedure. There's no need to wait, as composite fillings harden right away when exposed to a curing light.

5. Is it normal to feel sensitive after a silver filling and for how long?

Code A: It is normal to feel sensitivity after a silver filling, especially to hot and cold, for a few weeks. The depth of the cavity removed can affect the level of sensitivity experienced. If the sensitivity persists beyond a few weeks or gets worse, it is advisable to contact your dentist for further evaluation.

Code B: It's common to experience some sensitivity to hot and cold temperatures after a silver filling (amalgam) is placed. This sensitivity usually lasts a few days to a week. If it persists or worsens, contact your dentist.

6. I was having almonds yesterday and suddenly pain started in my upper left tooth, what could be the reason?

Code A: Sorry, I am not familiar with that topic. Please contact Aga Khan University helpline.

Code B: Pain while eating almonds could be due to various dental issues, such as a cracked tooth, cavity, or previous dental work. It's best to consult your dentist for a thorough examination and diagnosis.

7. I have had pain and swelling after I got my root canal treatment initiated yesterday, what is the plan of action?

Code A: If you are experiencing pain and swelling after your root canal treatment, it is important to contact your dentist or endodontist immediately. They may prescribe additional medication, such as antibiotics, and may ask you to come in for further treatment. It is crucial to follow their instructions for proper care and management.

Code B: Pain and swelling after a root canal treatment are not uncommon. It's typically managed with over-the-counter pain relievers and should improve within a few days. If the pain is severe or persists, contact your dentist for further evaluation and guidance.

8. The tooth hurts on biting ever since I got my filling done, what could be the possible reason?

Code A: If your tooth hurts on biting after getting a filling, it could indicate that the filling is not properly aligned with your bite. It is recommended to contact your dentist for a quick adjustment to the filling. Ignoring this issue may lead to further irritation and a toothache.

Code B: Pain while biting after a filling could indicate an issue with the filling itself, like an uneven bite or improper shaping. Contact your dentist to have it evaluated and adjusted to alleviate the discomfort.

9. My tooth was sensitive when the filling was done but now it hurts so much that I require painkillers for that, what to do?

Code A: If your tooth was sensitive when the filling was done but now it is causing severe pain that requires painkillers, it is important to contact your dentist. They will evaluate the situation and may recommend further treatment or adjustments to the filling. It is crucial to address the issue to alleviate the pain and ensure proper healing.

Code B: Increased sensitivity and pain after a filling may be due to an underlying issue. It's important to consult your dentist for a thorough examination to determine the cause and plan appropriate treatment. Relying on painkillers alone is not a long-term solution.

10. How long should I wait before I get a crown for my root canal-treated tooth?

Code A: After a root canal treatment, it is important to get a crown placed on your tooth as soon as possible. Delaying the placement of the crown may increase the risk of tooth fracture or possible loss of the tooth. It is recommended to schedule an appointment with your dentist to have the permanent crown placed within a month after the root canal treatment.

Code B: Typically, a dental crown is placed on a root canal-treated tooth within a few weeks to protect and strengthen it. Your dentist will provide specific guidance based on your case.

Fixed and Removable Prosthodontics

1. When will my speech get better with the complete denture?

Code A: Your speech will improve with the complete denture over time. In the first few days, your speech may be altered, and you may produce extra saliva. However, with practice and time, your speech will gradually return to normal. Reading out loud for 5 to 10 minutes a day can also help improve your speech.

Code B: Improvement in speech with complete dentures varies among individuals. It may take a few weeks to adapt to the new dentures and regain natural speech. Practicing speaking and wearing the dentures regularly can help expedite the process.

2. Can I eat normally after getting a pair of upper and lower dentures?

Code A: After getting a pair of upper and lower dentures, you may need some time to adjust to eating with them. Start with soft foods and gradually introduce harder foods as you become more comfortable. Avoid very hard or sticky foods that may dislodge the dentures.

Code B: You can eat normally with upper and lower dentures, but it may take some time to adapt to them. Start with soft foods, then gradually reintroduce your regular diet as you become accustomed to the dentures. Patience and practice are key for comfortable eating.

3. Would the denture teeth generate biting force just like the natural teeth?

Code A: Yes, denture teeth are designed to generate biting force similar to natural teeth. However, it may take some time to adjust to the dentures and regain full biting force. It is important to follow proper denture care and maintenance to ensure optimal function and longevity of the denture teeth.

Code B: Denture teeth may not generate the same biting force as natural teeth. It might take some adjustment to get used to dentures. Dentures are designed to function well, but they won't be as strong as your natural teeth.

4. My denture is getting loose day by day, what to do?

Code A: If your denture is becoming loose day by day, it is important to contact your dentist for an evaluation. Loose dentures can be caused by changes in the underlying bone structure or changes in the fit of the denture over time. Your dentist may recommend adjustments, relining, or possibly a new denture to improve the fit and stability. Avoid using over-the-counter adhesives without consulting your dentist, as they may mask underlying issues.

Code B: If your dentures are becoming loose, it may be due to changes in your jawbone. See your dentist for an adjustment or to discuss potential options like relining or replacing the dentures to ensure a better fit.

5. The denture wire is hurting me, can I fix it myself?

Code A: It is not recommended to try fixing the denture wire yourself if it is causing discomfort or pain. Adjusting or repairing dentures should be done by a dental professional who has the necessary skills and tools. Contact your dentist to schedule an appointment and have them assess and address the issue with the denture wire. Attempting to fix it yourself may lead to further damage or discomfort.

Code B: It's not recommended to try fixing denture wires yourself. Any adjustments or repairs should be done by a dental professional to ensure the dentures remain functional and comfortable. Contact your dentist for assistance.

6. I have an ulcer from the denture, should I continue wearing dentures?

Code A: If you have an ulcer from the denture, it is advisable to temporarily stop wearing the dentures until the ulcer heals. It is important to keep the surgical site clean and free of food particles. Please consult your dentist for further guidance.

Code B: If you have an ulcer from your dentures, it's best to stop wearing them and consult your dentist. They can assess the issue, adjust the dentures, and provide guidance on when it's safe to resume wearing them.

7. Is there a need to store dentures in hot boiling water overnight?

Code A: No, it is not necessary to store dentures in hot boiling water overnight. Dentures should be stored in a denture-cleaning solution or plain water when not in use. Boiling water can cause damage to the dentures and should be avoided. It is important to follow the specific instructions provided by your dentist for denture care.

Code B: No, its not recommended to store dentures in hot boiling water overnight. Dentures should be kept in a denture-cleaning solution or plain water when not in use. Boiling water can damage dentures or cause them to lose their shape.

8. My temporary crown got dislodged, do I have to worry about it?

Code A: If your temporary crown gets dislodged, it is important to contact your dentist as soon as possible. They will be able to advise you on whether it needs to be recemented or repaired. Leaving the temporary crown dislodged for an extended period of time may result in complications.

Code B: A dislodged temporary crown should be addressed promptly. Contact your dentist for re-cementing or replacement to protect the prepared tooth and ensure the success of the permanent crown placement.

9. For how long do I avoid sticky food after permanent crown cementation?

Code A: After the permanent crown is cemented, it is advised to avoid sticky foods for the first 24 hours. After that, you may eat, drink, and clean your tooth as you normally would. However, if your bite feels uneven or if you experience any discomfort, it is recommended to contact your dentist for further evaluation.

Code B: Avoid sticky foods for a few days after permanent crown cementation to allow the cement to fully set. Your dentist may provide specific instructions based on your case.

10. Is there a need to store dentures in hot boiling water overnight?

Code A: No, it is not necessary to store dentures in hot boiling water overnight. Dentures should be stored in a denture-cleaning solution or plain water when not in use. Boiling water can cause damage to the dentures and should be avoided. It is important to follow the specific instructions provided by your dentist for denture care.

Code B: No, it's not recommended to store dentures in hot boiling water overnight. Dentures should be kept in a denture-cleaning solution or plain water when not in use. Boiling water can damage dentures or cause them to lose their shape.
