# Supplementary material for: Leveraging Large Language Models in the delivery of post-operative dental care: a comparison between an embedded GPT model and ChatGPT
Source: BDJ Open. 2024 Jun 12;10:48. doi: 10.1038/s41405-024-00226-3 (PMC11169374; doi:10.1038/s41405-024-00226-3)
Supplement: Supplementary file 7 — Description of Additional Supplementary Files [file 41405_2024_226_MOESM7_ESM.pdf]

Description of Additional Supplementary Files

Supplementary Note 1. Dental post-operative instructions.

Supplementary Note 2. Questions and responses generated via both models for four different specialties of dentistry.

Supplementary Note 3. CVI-AI-based post op education: oral and maxillofacial surgery.

Supplementary Note 4. CVI-AI-based post op education: operative dentistry.

Supplementary Note 5. CVI-AI-based post op education: prosthodontics.

Supplementary Note 6. CVI-AI-based post op education: peridontics.
